# High species richness and turnover of vascular epiphytes is associated with water availability along the elevation gradient of Volcán Maderas, Nicaragua

**DOI:** 10.1002/ece3.9501

**Published:** 2022-11-22

**Authors:** Hazel K. Berrios, Indiana Coronado, Travis D. Marsico

**Affiliations:** ^1^ Department of Biological Sciences Arkansas State University, State University Jonesboro Arkansas USA; ^2^ Universidad Nacional Autónoma de Nicaragua (UNAN‐León) León Nicaragua

**Keywords:** climate, diversity, elevation, epiphyte, fern, generalized dissimilarity model, gradient, orchid, rarefaction, species richness, tropical forest, vascular plant

## Abstract

Research that has been conducted documenting species richness patterns on tropical mountains has resulted in conflicting observations: monotonic declines with increasing elevation, monotonic increases with increasing elevation, and a mid‐elevation “bulge.” Currently, it is unclear if these differences are due to environmental differences among study areas, the taxonomic groups or ecological groups (e.g., growth form) sampled, or the scale of study along elevation gradients. Because of the difficulty in sampling and identifying canopy‐dwelling plants, the number of inventories quantifying tropical epiphytes is relatively limited and recent. In this study, we provide a detailed qualitative and quantitative assessment of the vascular epiphyte flora and its spatial distribution on Volcán Maderas, Isla de Ometepe, Nicaragua, including weather and environmental measurements along the entire elevation gradient of the volcano. We sampled epiphytes in five distinct forest types associated with increasing elevation as follows: dry forest, humid forest, wet forest, cloud forest, and elfin forest. Five weather stations were placed along the elevation gradient for us to relate observed patterns to environmental conditions. A mid‐elevation peak in species richness was detected for all vascular epiphytes at approximately 1000 m in elevation (cloud forest), yet epiphyte abundance increased with increasing elevation. In total we identified 206 taxa of vascular epiphytes belonging to 26 families and 73 genera. The most species‐rich family was the Orchidaceae with 55 species for the entire elevation gradient, followed by Bromeliaceae (29 species), Araceae (23), Polypodiaceae (25), Dryopteridaceae (16), and Piperaceae (11), with all other families represented by fewer than 10 species each. We found that richness patterns differ phylogenetically across epiphyte groups, possibly due to different adaptive strategies, and species for the most part appear to be narrowly distributed within specific habitat zones along the elevation gradient. Variables associated with moisture, precipitation, humidity, mist, or cloud cover are key to understanding the observed patterns.

## Graphical abstract

We documented and examined patterns of species richness and abundance of tropical epiphytes along the entire elevational gradient of an isolated, island volcano. Our complete and detailed sampling along the gradient improves our understanding of vascular epiphyte distribution and its association to weather variables, based on direct observations. Our results demonstrate that the mid‐domain effect is real for these epiphytes and that patterns of epiphytes differ phylogenetically within epiphytic vascular plants and that species turnover is high along the elevational gradient, showing little overlap of species even among adjacent forests.

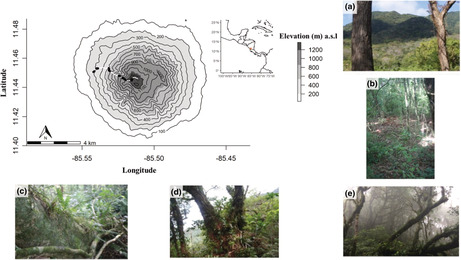

## 1 INTRODUCTION

Montane elevation gradients are important for understanding biogeographic patterns that result from ecological and evolutionary process (Lomolino, 2001). Moreover, it is important to understand patterns of biodiversity in Neotropical montane forests because these forests are important reservoirs of global biodiversity and ecosystem services (Nadkarni & Longino, 1990; Nadkarni & Matelson, 1989; Yanoviak et al., 2007). For example, Neotropical forests contain the greatest number of plant species on Earth (Raven et al., 2020), and epiphytes contribute substantially to this diversity by comprising 10% of all global vascular plant species (Zotz et al., 2021). In particular, epiphytes contribute to numerous ecological processes (Gotsch et al., 2016) and significantly contribute to regional plant species richness (Cardelús et al., 2006; Gentry & Dodson, 1987). Yet tropical montane forests are threatened by human activities, including deforestation, over‐exploitation of other resources, climate change, and an increasing threat of species invasion (Foster, 2001; Hu & Riveros‐Iregui, 2016; Lawton et al., 2001; Oliveira et al., 2014).

Botanical research documenting species richness patterns on tropical mountains has resulted in three major observations: monotonic declines with increasing elevation, monotonic increases with increasing elevation, and a mid‐elevation “bulge.” Monotonic declines in species richness with elevation are particularly prominent on large elevation gradients (greater than 1000 m a.s.l.), and have been attributed to cold, harsh alpine conditions near the summits of tall mountains that reduce plant species richness with increasing elevation. For example, monotonic declines have been shown for all vascular plants sampled in Cerro Grande in Jalisco, Mexico (2500 m a.s.l.; Vazquez & Givnish, 1998), in the mountains of the Bolivian Andes (3950 m a.s.l.; Kessler, 2002), and in Mount Kinabalu, Borneo (4094 m a.s.l.; Grytnes & Beaman, 2006 [weak hump‐shaped distribution]). Additionally, monotonic declines have also been detected for specific plant growth forms such as liana species richness patterns in Chile (1100 m a.s.l.) and New Zealand (1220 m a.s.l.; Jiménez‐Castillo et al., 2007), and trees in Borneo (1972 m a.s.l.; Slik et al., 2009) and in the Cordillera El Consuelo in the Andes of southern Ecuador (2600 m a.s.l.; Homeier et al., 2010).

Monotonic increases with increasing elevation have been attributed to the association between specific taxonomic groups and environmental factors. For example, fern species richness patterns show a monotonic increase with increasing elevation in the Island of Tobago and in Peninsular Malaysia, and this pattern was explained by climatic factors (Kessler et al., 2011). In light of different biogeographic patterns, some authors have questioned the generality of findings, indicating that decreases or increases in richness with increasing elevation may apply to specific taxonomic or ecological groups (e.g., different growth forms) or because research did not investigate the entire elevation gradient (Nogués‐Bravo et al., 2008; Rahbek, 2005). In fact, Rahbek (2005) showed that monotonic trends along elevational gradients were not the predominant pattern; instead, 80% of the studies describing species richness patterns along elevational gradient recorded a mid‐elevation peak (or hump‐shaped curve) when biases such as incomplete sampling along the gradient were considered.

Multiple studies in Neotropical systems have shown that epiphyte species richness peaks at the mid‐elevations along montane gradients (Cardelús et al., 2006; Colwell & Lees, 2000; Grytnes & Vetaas, 2002; Watkins et al., 2006; Wolf & Flamenco, 2003), and the mid‐domain effect, whereby low‐elevation and high‐elevation specialists overlap with mid‐elevation taxa, has support from multiple studies. For example, Krömer et al. (2005) recorded a mid‐elevation peak of vascular epiphyte richness from the Andes of Bolivia. In this study, the highest elevation areas were also distinct in species composition from the lowest elevation areas because high elevations contained the highest percentage of pteridophytes and the lower elevations contained a higher percentage of species in the Araceae and Orchidaceae families. The middle elevations contained all five plant groups that were recorded at the lower and higher elevations. Krömer et al. (2005) noted that the diversity patterns of vascular epiphytes from lowland to mid‐elevations were associated with rainfall: the higher the rainfall levels, measured as mean annual precipitation, the higher the species richness. In a study in Costa Rica, the mid‐elevation peak was found and also attributed to precipitation (Cardelús et al., 2006). However, in another study from Chiapas, Mexico, although a mid‐domain diversity peak was observed, elevational diversity patterns did not correlate with rainfall (Wolf & Flamenco, 2003).

Because of the difficulty in sampling and identifying canopy‐dwelling plants, the number of inventories quantifying tropical montane epiphytes is relatively limited and recent, resulting in a substantial gap in knowledge, even though these forest canopies contain significant biodiversity and forest biomass (Lowman & Nadkarni, 1995). For example, in Ecuador 27.8% of the total floristic richness corresponds to epiphytes and hemiepiphytes (Jørgensen & León‐Yánez, 1999), and this value increases to 65% of the total flora in montane rain forests of Venezuela (Kelly et al., 1994). In the cloud forests of Costa Rica, over 80% of the total forest biomass belongs to non‐woody canopy plants (Nadkarni et al., 2004). In the case of Volcán Maderas, multiple ecological and taxonomic research initiatives have been conducted (Gilléspie & Martínez Sánchez, 1995; Huettmann, 2015). However, for vascular epiphytes, georeferenced specific spatial descriptions were lacking and a standardized effort for documenting species richness was non‐existent.

We provide a detailed qualitative and quantitative assessment of the vascular epiphyte flora and its spatial distribution. In particular, we investigated species turnover, here defined as the amount of change in species composition along an elevation gradient. We also aimed to understand how species turnover is affected by climatic factors.

## 2 MATERIAL AND METHODS

### 2.1 Study area

**FIGURE 1:**
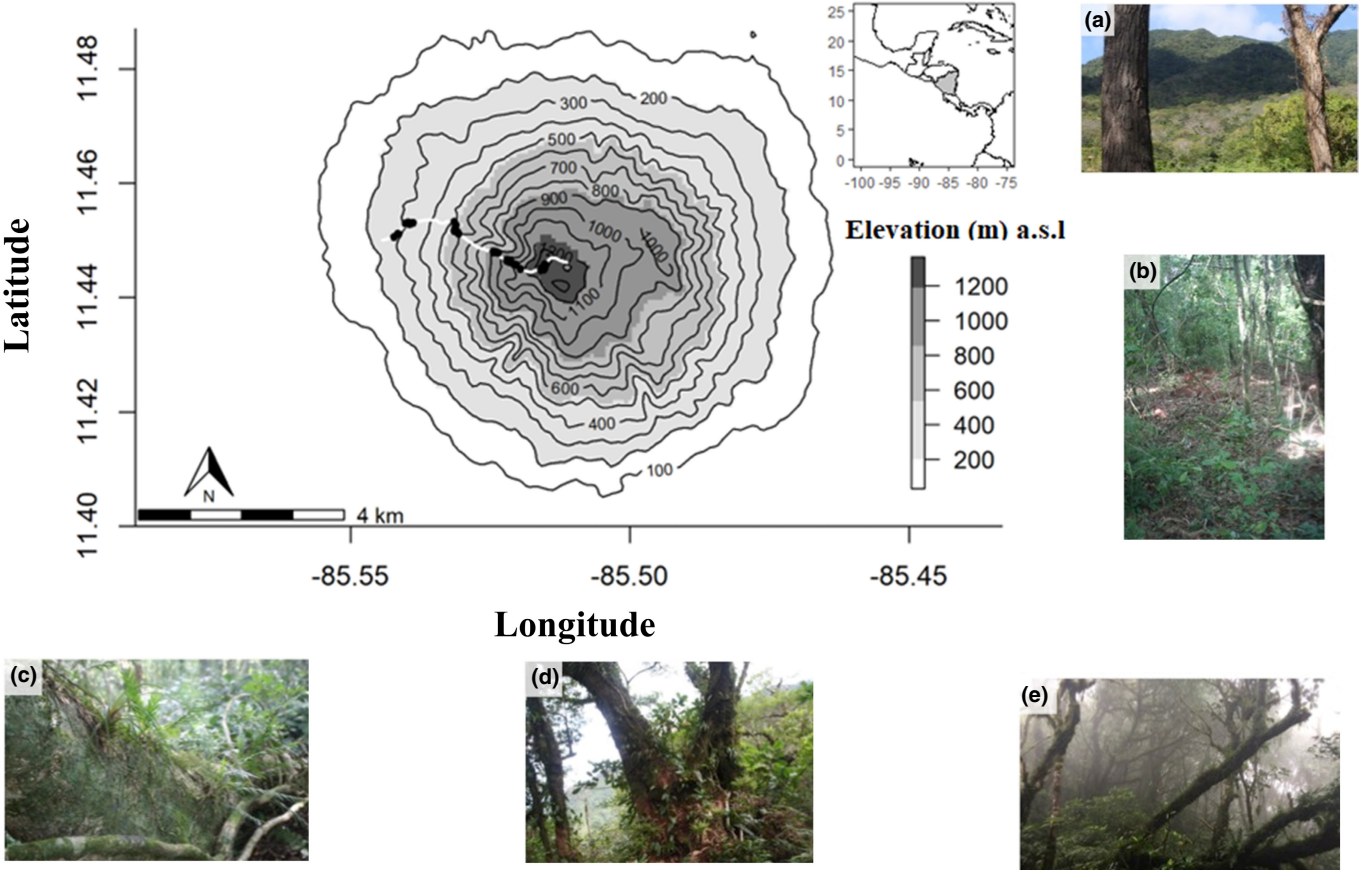
Sampling locations along the elevation gradient of Volcán Maderas, Nicaragua. The white line represents the Merida Trail. Each photograph depicts a forest type as follows: (a) Dry forest (50–400 m): seasonally deciduous vegetation and high mean temperatures (~26°C); (b) Humid forest (401–700 m): well‐developed shrub layer of native bamboo and a high diversity of hemiepiphytes; (c) Wet forest (701–1000 m): permanent damp‐wet soils; (d) Cloud forest (1001–1060 m): high humidity and cloud cover; and (e) Elfin forest (1301–1394 m): high winds and stunted trees.

We studied in one of the most diverse forests in Nicaragua with high conservation value on Isla de Ometepe, an island located in the largest freshwater lake of Central America, Lake Nicaragua (Colcibolca; Figure 1). Ometepe comes from the Nahuatl word meaning “two peaks” referring to its two volcanoes Concepción and Maderas. Volcán Maderas (11°26′44″N, 85°30′54″W) is a dormant stratovolcano that contains a multi‐ecosystem forest of biodiverse habitats with a high proportion of rare species that is formed by the persistent orographic cloud formation near the summit of the volcano (Borgia & van Wyk de Vries, 2003). At Volcán Maderas, the local climate is affected by land use, ocean‐driven humidity (prevailing trade winds from the Caribbean), volcanic history, and unique topography (Borgia & van Wyk de Vries, 2003). Volcán Maderas is a small sub‐conical volcano with steep flanks (Grosse et al., 2009), with a maximum elevation of 1394 m a.s.l., and a diameter of ~10 km (Borgia & van Wyk de Vries, 2003). Last volcanic activity occurred ~60,000 years ago and therefore future volcanic eruptions from Maderas are not considered an immediate threat to the island. Instead, mudslides caused by heavy rainfall events are of primary concern (Kapelanczyk et al., 2012).

Three distinct old‐growth forest types have been described for Volcán Maderas (Gilléspie & Martínez Sánchez, 1995): Humid forest (500–800 m), wet forest (800–1100 m), and elfin forest (1100 m to the summit of the volcano). For a universal comparison, these forests fit under the pre‐montane and lower montane elevational category based on the Holdridge life zones (Holdridge, 1967). In this study, our sampling and forest type classification was more specific than that of Gilléspie and Martínez Sánchez (1995). We extended our sampling to the lower areas of the volcano (lower than 500 m), which we primarily classified as a dry forest, due to the prominence of intense seasonality of precipitation and preponderance of drought‐deciduous trees. The dry forest habitat ranged from the base of the volcano (at ~50 m a.s.l.) to about 400 m a.s.l. In addition to including dry forest at the lower elevations, we modified the habitat ranges as follows: a lower elevation start to the wet forest (701 m), a higher elevation start to the elfin forest (1301 m), and the addition of the cloud forest (1001–1300 m). The inclusion of the cloud forest habitat was needed because it is a forest type often shrouded in clouds, but with taller trees than what is generally recognized as elfin or dwarf forest. Therefore, our surveys were conducted in five distinct forest types associated with increasing elevation as follows: Dry forest (50–400 m), humid forest (401–700 m), wet forest (701–1000 m), cloud forest (1001–1300 m), and elfin forest (1301 m to the summit 1394 m), loosely following forest types described by Gilléspie and Martínez Sánchez (1995).

### 2.2 Environmental variables

We deployed five HOBO weather microstations (Onset Computer Corporation), one in each forest type, that measured temperature (°C), relative humidity (%), leaf wetness (%), and rainfall (mm). Instantaneous data were recorded every 15 min, for a total of 96 measurements/day. We calculated daily mean (totals for precipitation), minimum, and maximum readings for each forest type. The weather data were collected March 15, 2017 to May 22, 2017, which coincides with the transition from the dry season to the wet season in this part of Nicaragua. For all weather data descriptions see Figures S1–S4.

### 2.3 Plant abundance data, identification, and specimen collection

Along the Mérida Trail on the west slope of the volcano, we selected eight mature canopy trees from each of the five forest habitats (40 trees total; Figure 1). All eight trees in each forest type were sampled within 1 ha along the elevational gradient of the volcano, and we describe this sampling protocol as standardized sampling (ss; Figure 1). To capture complete epiphyte richness, we selected trees representative of their elevation. Canopy sampling consisted of recording richness and abundance data from the tree trunk and two lowest main branches (north and south). In order to maximize and record the most accurate species richness of vascular epiphytes per habitat, the visual tree selection consisted of the following guidelines as recommended by Gradstein et al. (2003): we avoided selecting trees in close proximity (~25 m). The oldest or largest trees were preferred, as well as trees with various bark characteristics and canopy structure, for example, branch diameter and structure (oblique vs. horizontal branches). Lastly, we preferred sampling different species of trees, where at least half of the selected trees per habitat were of different species.

All the ss trees were intensely photographed and videoed on the sampled branches. Eighty percent (32/40) of the trees were surveyed from the ground on a slope for easier visualization, using binoculars or through a high‐resolution camera with a zoom feature (Panasonic Lumix DMC‐FZ70 16.1 MP Digital Camera with 60× Optical Image), and 20% (8/40) of the trees were free‐climbed for direct sampling of vouchers. There are some limitations to sampling epiphytes from the ground, including the possibility of not observing relatively small plants species on top of branches such as those belonging to the family Selaginellaceae. Our approach using a high‐resolution camera with high‐powered zoom capabilities, however, allowed for the acquisition of data that were evaluated with greater time in the laboratory and were checked for correctness by multiple researchers, resulting in detailed and reproducible data collection. Plant abundance was determined only on Ss trees and was calculated by the number of individuals within an epiphyte species recorded from examining photographs and/or videos. For all hemiepiphytes, each stem attached to the tree was recorded as a separate individual; although this approach may overestimate abundance, it ensured a constant sampling effort across our study sites.

In addition to sampling and recording data on selected trees (Ss), plant specimens were collected from fallen branches opportunistically (opportunistic sampling [Os]) to capture a more complete species richness of canopy epiphytes in each forest type and to ensure we collected vouchers for species‐level identification of epiphytes in the sampled communities.

Voucher specimens were deposited in Miguel Ramirez Goyena UNAN‐León Herbarium (HULE), with duplicates sent to the Arkansas State University Herbarium (STAR). Plant identifications were determined in the STAR Herbarium using collected mature or flowering specimens. All pteridophyte (ferns and their allies) identifications were determined using Flora de Nicaragua Tomo IV Helechos (Gómez & Arbeláez, 2009), supplemented with image and data searches online, and all flowering plants were determined using the online dichotomous key and plant descriptions from the Flora de Nicaragua (Tropicos, 2018; updated and based on Stevens, Ulloa, Pool, & Montiel, 2001, Tomos I, II, III) and various keys from neighboring Costa Rica (Hammel et al., 2003, 2004, 2007, 2010).

### 2.4 Data analyses

Hemiepiphytes were included in the analyses with epiphytes. Species richness curves for each forest type were determined using the number of species per site from sampled trees (Ss), fallen branches (Os), and the sum from both methods (Ss + Os). Species richness was also calculated for the main taxonomic groups of vascular epiphytes (families Orchidaceae, Bromeliaceae, Piperaceae, Araceae, and Division Pteridophyta [seedless vascular plants that include the families: Aspleniaceae, Blechnaceae, Dryopteridaceae, Gleicheniaceae, Hymenophyllaceae, Lomoriopsidaceae, Lycopodiaceae, Lygodiaceae, Oleandraceae, Ophioglossaceae, Polypodiaceae, Pteridaceae, Sellaginellaceae, Tectariaceae, Thelypteridaceae, and Vittariaceae] and others [plant families with fewer species in each family: Begoniaceae, Cactaceae, Clusiaceae, Melastomaceae, and Passifloraceae]). Species richness was used to detect the elevational distribution pattern and determine the relative contribution of each of the predominant taxonomic groups to forest type. Juvenile plants were excluded from the calculation as many of their identifications were uncertain. In addition, we examined species richness relationships with the environmental variables by calculating the correlation coefficient among the variables using the “corrplot” package in R (Wei & Simko, 2021).

To determine the completeness of our sampling method, rarefaction curves were generated using the *iNEXT* function in the “iNEXT” package in R (Hsieh et al., 2020). This analysis only included data from trees sampled (Ss) as abundance data were not recorded from opportunistic samples. We used the estimated asymptote of each forest type to compare the species richness between them and to discard the possibility that differences among sites were attributed to sample size.

To better understand how the main environmental factors may drive species composition patterns along the elevational gradient, we performed a generalized dissimilarity model (GDM) using only the Ss samples, which is a regression method that explains changes in species composition under a changing environment or along environmental gradients (Ferrier et al., 2007). GDM models species compositional turnover across pairs of sample sites (i.e., beta diversity) as a function of their environmental differences (Ferrier et al., 1999). The function *formatsitepair* in the “gdm” package (Manion et al., 2018) was used to compute the dissimilarity values among pairs of sites. We constructed a site‐pair table containing dissimilarity values in the first column and the environmental predictors of a given site, *x*, with those of a second site, *y*, in the remaining columns. The dissimilarity values computed by the GDM used the Bray–Curtis Index (Bray & Curtis, 1957). To remove multicollinearity and facilitate better interpretation of the GDM analysis, climate variables that were highly collinear with each other were removed (*R* ^2^ > 0.7) using the *cor* function in the “caret” package (Kuhn et al., 2018). The GDM was then fit using our two resulting predictors (Rainfall and Leaf Wetness) to our response variable (species composition distance values) for each site pair and created monotonically increasing functions for each environmental factor using a Generalized Linear Model.

Lastly, we ran the hierarchical clustering analysis using the *hclust* function with (method = “average”) in the R software (R Core Team, 2018) version 3.5.0. This analysis used the dissimilarity values derived from the beta diversity between pairs of sites to construct each cluster from a bottom‐up approach. That is, the first step consisted of a cluster that contained the lowest dissimilarity values between a pair of sites, and a second cluster was constructed by computing the distance between the first cluster and another site. This step was continued until all sites were merged into a single cluster. The GDM and hierarchical clustering analysis were only performed using the Ss dataset.

## 3 RESULTS

### 3.1 Weather patterns among the five forest types along an elevation gradient of Volcán Maderas

The weather measurements collected for each forest type from March to May represent the transition between the dry and the wet seasons, with April being the hottest month of the year. The dry forest is characterized by deciduous trees, a lack of cloud cover, low wind speeds, and high mean temperatures (~26°C), resulting in relatively dry soils (Figures S1a and S2). The humid forest is characterized by lower values for temperature and higher humidity levels than the dry forest (Figure S1a,c,d). Despite the lower temperature readings and changes in vegetation composition, weather readings of the humid forest were more like those of the dry forest than to the rest of the other forest types (Figures S1–S4). This is particularly pronounced when comparing maximum temperature and leaf wetness readings between the dry forest and the humid forest, where maximum values for both habitats overlapped (Figure S2a,c).

Rain events were sporadic throughout mid‐March to the start of May, with only four major rain events occurring in 45 days (Figure S1b). During the peak of the dry season (March–early April) on Volcán Maderas, vascular epiphytes were exposed to drought‐like conditions for a minimum of 11 days with no measurable rain. We observed a pattern of a higher frequency of rain events as the dry season transitioned into the rainy season (Figure S1b). Increased rain event frequency was a main feature of all forest types later in the recording period (late April–May) as the rainy season approached (Figure S1b).

With increasing elevation, the role of cloud water deposition into the surrounding vegetation clearly plays a main role in ameliorating the drought effects with increasing mean relative humidity and leaf wetness levels (Figure S1c,d). For example, even in the absence of rain between March and April, mean leaf wetness values for the higher elevations remained above 25% (Figure S1c) and humidity levels remained above 75% humidity (Figure S1d). It is important to note that when analyzing only the minimum reading values throughout the days, relative humidity recorded during the peak of the dry season, at the end of March, shows readings as low as 45% humidity for all forest types (Figure S3d).

The wet forest showed lower mean temperatures than the dry forest or humid forest, which clearly separates this habitat from the lower elevation ones by four degrees, with mean temperatures averaging 21°C in March–May (Figure S1a). The mean temperature for the cloud forest is approximately 20°C, and the elfin forest at the summit of the volcano was the coolest forest type (mean 18°C; Figure S1a).

Daily weather fluctuation analysis for Volcán Maderas, shows a conspicuous peak for temperature for all forest types from 13:00 to 16:00 (Figure S4a,c,d). Interestingly, the daily temperature patterns follow those expected by decreasing with increasing elevation, except that the peak temperature at our second‐lowest elevation site is a higher temperature and slightly later in the day than at the lowest elevation (Figure S4d). This result could be due to the more open forest canopy at the placement site of the weather‐recording equipment. Leaf wetness for all forest types decreases during the hottest period each day (Figure S4c,d). Even with increasing temperature throughout the day, relative humidity for the highest elevation area remained relatively constant, at around 98% (Figure S4d). In contrast, the lower elevation areas showed marked, decreasing relative humidity values to as low as 72% (Figure S4d). Most rainfall periods occurred early in the mornings and evenings/nights from 05:00 to 08:00 and from 17:00 to 01:00 and were less frequent during the hottest period of the day from 10:00 to 16:00.

### 3.2 Species richness and abundance patterns

**FIGURE 2:**
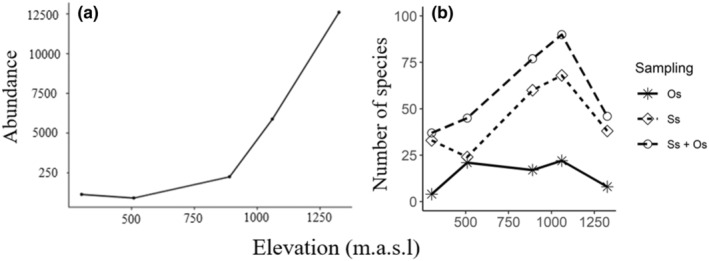
Elevational distribution of vascular epiphyte abundance (a) and species richness (b) on Volcán Maderas, Nicaragua. This figure is based on five sampling elevations; however, a line is shown to ease visualization of the pattern (geom_line in R version 3.5.1). Three lines are shown in panel B representing species sampled by ss, opportunistic sampling only (Os), and the sum of the two sampling strategies (ss + Os). 302 m = dry forest, 509 m = humid forest, 890 m = wet forest, 1060 m = cloud forest, and 1325 m = elfin forest.

From the 40 trees sampled (Ss), vascular plant identifications were made totaling 22,797 individual plants, representing 140 species across all forest types. On average, this resulted in 567 ± 276 SE individual epiphytes and hemiepiphytes per sampled tree, though abundances varied by forest type, with increased abundance with increased elevation (Figure 2a). The standardized observations (Ss) in combination with the collected specimens (Os) resulted in 190 identified species of vascular epiphytes belonging to 25 families and 73 genera (Tables S1 and S2). The most species‐rich family overall was the Orchidaceae with 54 species for the entire elevation gradient (Tables S1 and S2). The second‐most species‐rich family was the Bromeliaceae (29 species). The family with the third highest species richness was Araceae (20). The two most species‐rich pteridophyte families were Polypodiaceae (25) and Dryopteridaceae (14). Lastly, Piperaceae had 11 species, and all other families contained fewer than 10 species each. Of the 190 unique taxa, 64% were identified to species level, 17% were identified to the genus level only, and 18% contained unique morpho‐descriptions but were assigned to their respective plant families (Table S2). Morphospecies were particularly prevalent in the Araceae (40%) for which we had many sterile specimen observations and Orchidaceae (33%), which is notoriously difficult to identify and for which we also had many sterile specimen observations.

**FIGURE 3:**
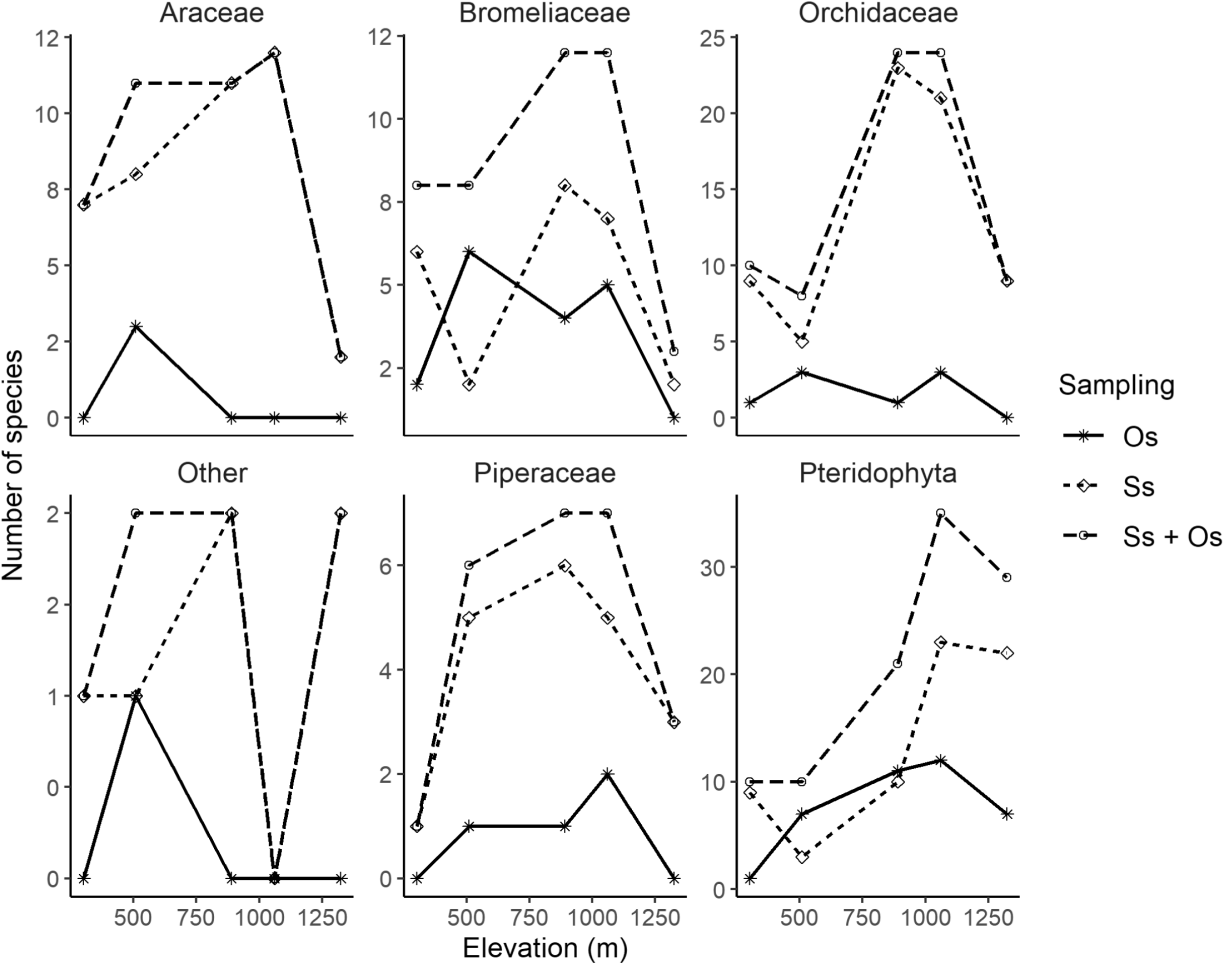
Species richness elevational distribution of the predominant families of vascular epiphytes in Volcán Maderas, Nicaragua. A line is shown to ease visualization of the pattern (geom_line in R version 3.5.1). Three lines are shown in each panel representing species sampled by ss, opportunistic sampling only (Os), and the sum of the two sampling strategies (ss + Os). 302 m = dry forest, 509 m = humid forest, 890 m = wet forest, 1060 m = cloud forest, and 1325 m = elfin forest.

The Ss method captured a unimodal pattern for the overall species richness with elevation (Figure 2b), and the Os method supplemented the species richness, but did not change the overall pattern of Ss. Epiphyte species richness was lowest in the dry forest with 37 total species of vascular epiphytes recorded (Figure 2b). The plant families with the highest species richness in the dry forest were Orchidaceae and Bromeliaceae (10 and 8 respectively), Araceae (7), and Polypodiaceae (4). The rest of the five families contained singletons or doubletons species each (Figure 3; Tables S1 and S2). The dry forest transitions into the humid forest around 400 m and continues to near 700 m in elevation. The total species richness recorded for the humid forest was 45 (Tables S1 and S2). Unlike the other forest types, the humid forest had a large abundance of vine and liana hemiepiphytes of 11 species (Figure 3; Tables S1 and S2). At a higher elevation than the humid forest is the wet forest with damp to wet soil and a distinct vegetation ranging from 701–1000 m. Species richness for the wet forest was the second highest from the whole elevational gradient with 77 total species, of which 24 belonged to the Orchidaceae family, followed by Bromeliaceae (12), Araceae (11), and Piperaceae (7), with the remaining families containing fewer than five species (Tables S1 and S2). The cloud forest elevation ranges from 1001 to 1300 m. Species richness peaked in this forest type with 90 total species (Figure 2b). Similar to the wet forest, orchids were predominant with 24 species. The other families in this forest type are listed in decreasing order of species richness: Bromeliaceae and Araceae (12 each), Dryopteridaceae and Polypodiaceae (10 each), Piperaceae (7), Hymenophyllaceae (6), and the rest of the families contained two species or fewer each (Tables S1 and S2). At the highest elevations of the volcano is the elfin forest starting at 1301 m to the summit (1394 m). The elfin forest had the third highest value in species richness (46; Figure 2b). The total species richness values by family were as follows: Polypodiaceae (11), Orchidaceae (9), Dryopteridaceae (5), Hymenophyllaceae (5), with the rest of the families containing four species or fewer each (Tables S1 and S2).

**FIGURE 4:**
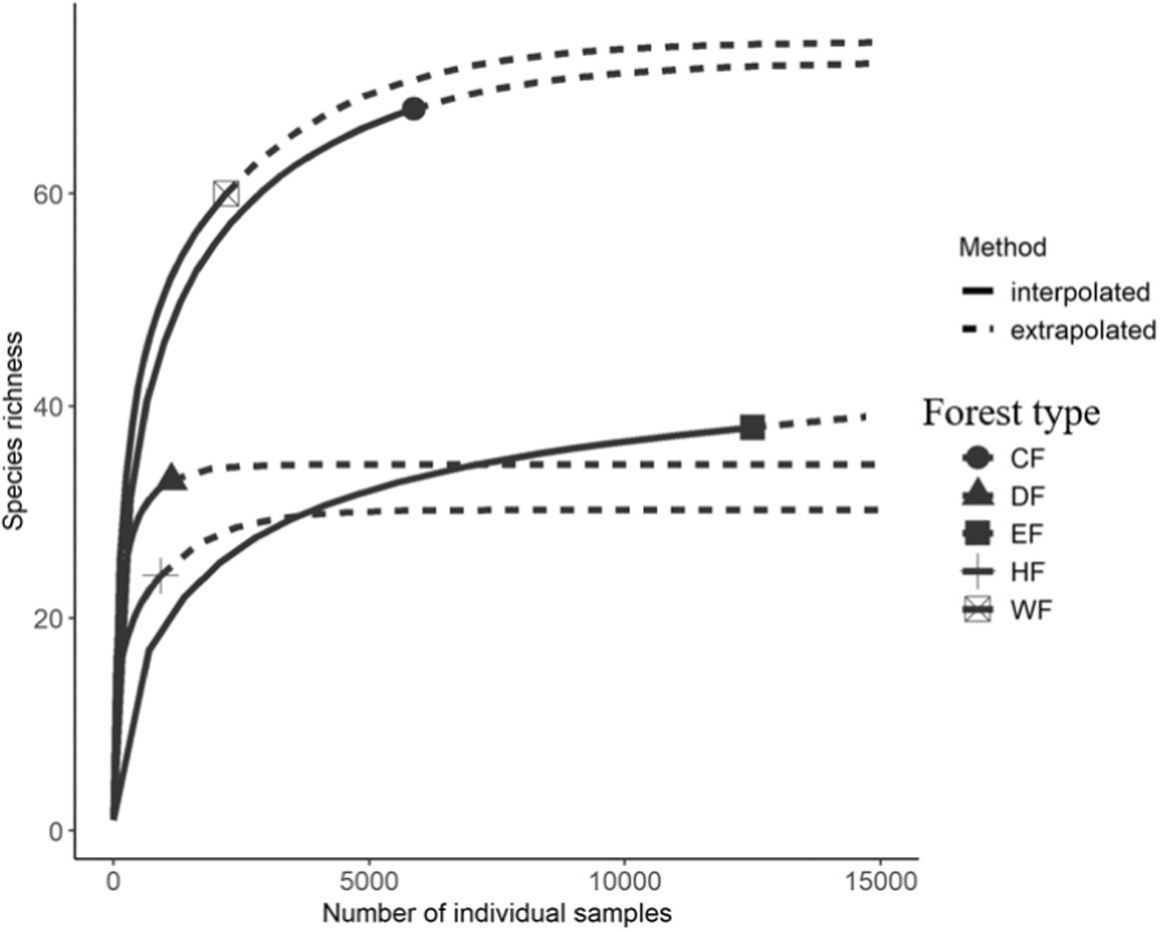
Rarefaction curves of vascular epiphytes species in Volcán Maderas, Nicaragua. DF = dry forest, HF = humid forest, WF = wet forest, CF = cloud forest, and EF = elfin forest.

Rarefaction analysis demonstrated that our sampling effort was sufficient to capture at least 80% of the species richness in all forest types of Volcán Maderas (Figure 4). For example, the elfin forest had an observed species richness of 38 and an estimated asymptotic species richness of 47. Similarly, the humid and wet forests were surveyed approximately to the estimated asymptotic species richness. The highest sampled species richness percentages were in the cloud forest and dry forest with 93% and 94% of the estimated species richness sampled for each forest type, respectively.

### 3.3 Important factors related to turnover among forest types

**FIGURE 5:**
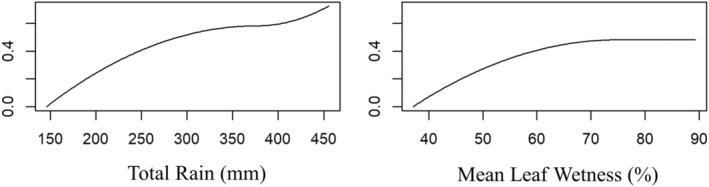
Fitted *I‐*spline partial functions (Ramsay, 1988) from the generalized dissimilarity modeling of vascular epiphyte assemblages using two environmental variables, total rainfall, and mean leaf wetness (MeanLW) of Volcán Maderas, Nicaragua.

It is impossible to disentangle the effects of our three moisture variables and our one temperature variable because they are highly intercorrelated, but all variables likely play a part, and moisture variables drive the species turnover patterns we observed. Temperature was, for example, highly negatively correlated with leaf wetness (i.e., higher elevations have lower temperature and higher leaf wetness; *r* = −0.98). After removal of highly collinear predictors (*R* ^2^ > 0.7; Table S3), only two predictors were considered in the species richness and turnover analyses, mean leaf wetness and total rainfall. Correlation analysis showed a negative correlation between species richness and rainfall, with a significant correlation coefficient of *r* = −0.23 and a significant positive relationship between species richness and leaf wetness of *r* = 0.57 (Figure 5).

The GDM indicated that both total rainfall and leaf wetness relate to patterns of beta diversity on Volcán Maderas. The GDM accounted for 43.91% of the deviance in observed turnover of vascular epiphytes. The maximum height and greater area under the curve for total rainfall indicate that the largest amount of turnover is observed along this gradient (Figure 5). Furthermore, the relationship between species turnover and each relevant environmental gradient varied (Figure 5). For example, greater epiphyte turnover was found between the range of 40%–70% leaf wetness and remained constant at higher values of leaf wetness (>70%; Figure 5). In contrast, species turnover was higher in both driest and wettest environments but remained relatively constant at elevations with intermediate rainfall (300 and 400 mm; Figure 5).

**FIGURE 6:**
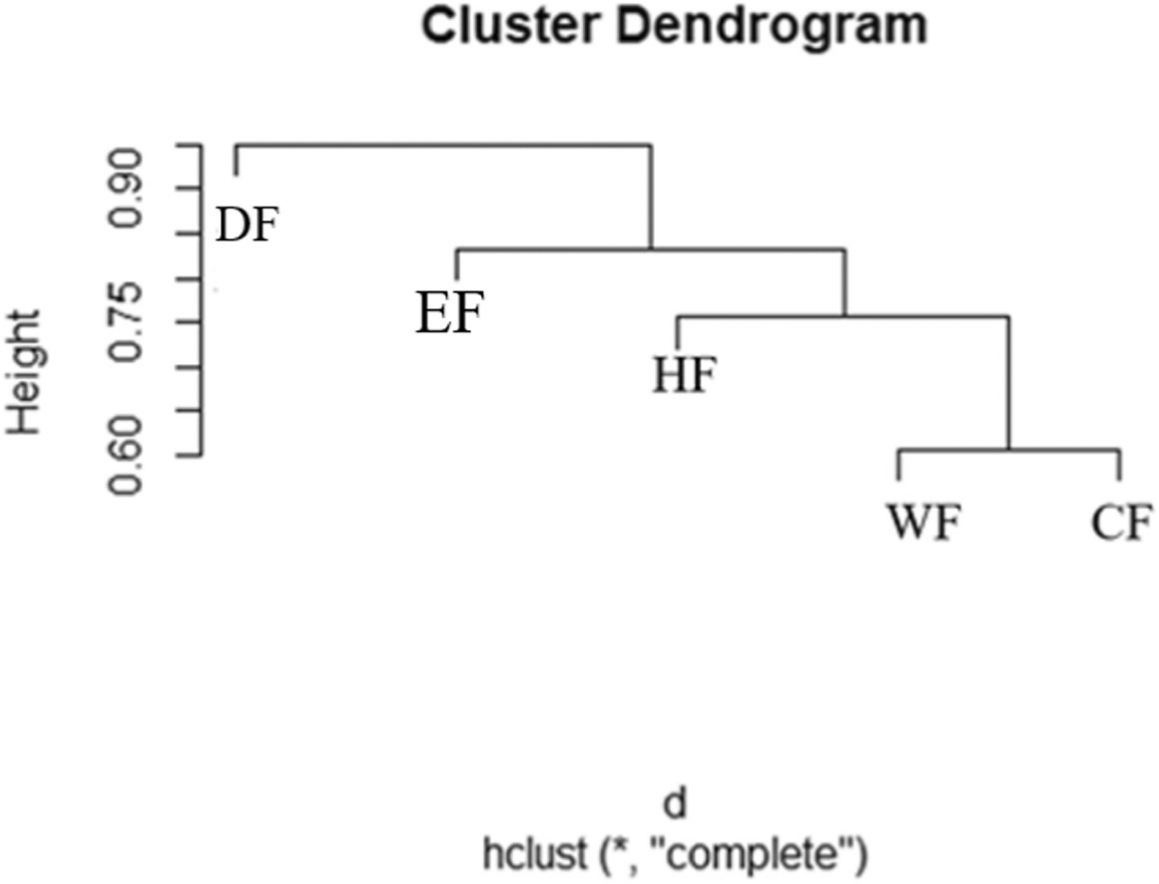
Hierarchical clustering analysis dendrogram for each forest type in Volcán Maderas. Height on the *y*‐axis indicates the dissimilarity value between clusters of observations “d” indicates distance or dissimilarity (as a clustering method) and hclust (“average”) indicates the linkage type used to perform the hierarchical clustering analysis (average dissimilarity linkage over all pairs). Elevations of each forest type are shown at the tips of the branches. Dry forest = 302 m, humid forest = 509 m, wet forest = 890 m, cloud forest = 1060 m, and elfin forest = 1325 m.

The forest types that are the most like each other in terms of their epiphytic species composition are the wet forest and cloud forest as shown by the formation of the first cluster with the lowest dissimilarity value of 0.60 (Figure 6). The humid forest is more similar to the wet forest and cloud forest than it is to the elfin forest (Figure 6). However, the elfin forest has a lower dissimilarity value between the rest of the forest types than the dry forest (Figure 6). The dry forest was most floristically distinct from all the other forest types (about 0.85; Figure 6). Another direct, and simpler, way of investigating species turnover across the forest types is to examine the number of unique species in each forest type (Table 1). Unique species are defined as species found in only one forest type. We observed a high percentage of unique species within each forest type; unique species represented close to half of the total species richness within each forest type (Table 1). This provides important evidence that species had narrow elevational distributions (see also Table S2).

**TABLE 1:** Number of species unique to each forest type

Forest type	Number of unique species (and percent of total species estimated by rarefaction^a^)
Dry forest	16 (46%)
Humid forest	15 (50%)
Wet forest	33 (45%)
Cloud forest	37 (51%)
Elfin forest	21 (45%)

^a^ Percentage calculations were rounded to the nearest whole number.

## 4 DISCUSSION

### 4.1 Patterns of biodiversity of canopy‐dwelling plants in tropical forests

In this study, we detected a mid‐elevation peak of the total species richness, at approximately 1000 m in elevation in the cloud forest (Figures 2 and 4). This pattern was consistent across taxonomic groups no matter the analytical approaches used. This was surprising given the relatively low maximum elevation of Volcán Maderas (1394 m). Other studies with similar elevational ranges to Volcán Maderas (≤1000 m) have detected a general monotonic relationship in species richness with elevation (noted by Rahbek, 2005). For example, Kessler et al. (2011) recorded a monotonic increase of species richness with increasing elevation for mountains peaks with similar heights to Volcán Maderas, and we expected this pattern as well because of drier habitats at the base of the volcano that are less conducive to epiphytic growth and a volcano summit that is never subjected to freezing temperatures. In contrast, a mid‐elevation peak of vascular epiphytes like we found has been demonstrated by multiple studies with markedly longer elevational gradients in tropical montane forest systems, and in these studies species richness peaks at elevations well above 1060 m at a local scale (Rahbek, 2005). For example, epiphyte species richness peaked at 1300 m in Hainan Island, China (Ding et al., 2016), 1430 m in Mexico (Hietz & Hietz‐Seifert, 1995), 1500 m in Bolivia (Krömer et al., 2005), and 1700 m in Colombia (Cleef et al., 1984; Wolf, 1994).

Unlike species richness, epiphyte abundance increased with elevation on Volcán Maderas, similar to what has been found for canopy epiphytes in Monteverde, Costa Rica (Gotsch et al., 2017). The prolonged exposure to the cloud water input seems to be responsible for the increased abundance of vascular epiphytes at the higher elevations (Ding et al., 2016; Gotsch et al., 2017), particularly in the elfin forest where abundance estimates double those from the cloud forest and are at least 11 times larger than those encountered in the dry and humid forests (Figure 2a). Yet, despite the elfin forest's higher abundance of epiphytes, rarefaction curves predict the elfin forest to have 40% fewer species than the cloud forest or wet forest (Figure 4), indicating that elfin forests harbor a dense mat of epiphytic vegetation comprising a species richness like the low elevation humid and dry forests, though not a similar species composition (see “Species Turnover” below and Tables S1 and S2).

Conflicting findings of species richness distribution patterns between studies that detected mid‐elevation peaks (Janzen, 1973; Lieberman et al., 1996; Whittaker, 1960; Whittaker & Niering, 1975) and studies that detected monotonic changes in species richness with elevation (Gentry, 1988; Hamilton, 1975; Kitayama, 1992; Odland & Birks, 1999; Vazquez & Givnish, 1998; Yoda, 1967) have been attributed to the role of “scale of analysis” (Rahbek & Graves, 2000). The importance of scale in determining the geographical patterns of species richness has been well documented (Hutchinson, 1953; Levin, 1992; Ricklefs, 1987; Schneider, 1994; Whittaker, 1977; Wiens, 1989). Rahbek (2005) provided an in‐depth review discussing the role of the size of the unit of sampling and the role of the geographical space covered. Many of the studies documenting a monotonic change in species richness did not fulfill the requirement of including data for the entire gradient being considered, thus potentially biasing results. When sampling effort was sufficient and samples from the entire gradient were recorded, a mid‐elevation peak of species richness dominated in the literature (Rahbek, 2005). Issues of scale could partly explain why even though Volcán Maderas is low in elevation in comparison with other tropical montane forests, our sampling effort in the entire gradient was sufficient to capture a hump‐shaped distribution.

Another possible explanation for Volcán Maderas indicating a mid‐elevation peak of vascular epiphytes could be attributed to possible tradeoffs between regional and local climate acting along the elevational gradient. For isolated island peaks like Volcán Maderas, a cloud forest can develop at substantially lower elevation than for continental mountain peaks presumably because of the exceptionally humid conditions that develop from the higher water vapor content of air that accumulates from the wind over open water (Bruijnzeel et al., 1993; Foster, 2001). Low‐elevation areas of the volcano such as the dry forest and humid forest are clearly and consistently distinguished from the high‐elevation areas in terms of climate (Figures S1–S4). In the high‐elevation areas, from the wet forest through the elfin forest, the role of cloud water deposition clearly plays a role in ameliorating the effects of drought during the peak of the dry season (Figure S1c,d), but this role diminishes at the low‐elevation areas. This constant input of moisture from the cloud condensation at high elevations creates a unique habitat for aerial vascular epiphytes that depend solely on atmospheric inputs for their survival. Although no consensus has been reached on the factors that determine the mid‐elevation peak for species richness of vascular epiphytes, our research and other studies have shown epiphyte richness to be positively associated with rainfall and humidity (Gentry & Dodson, 1987; Hemp, 2006).

### 4.2 Species turnover

We detected a high association between total rainfall and species turnover among the five forest types (Figure 5). High species turnover was demonstrated with rainfall at both the driest and wettest ends of the gradient, and more moderate turnover in mid‐elevation areas (Figure 5). Furthermore, total rainfall was inversely correlated with relative humidity (Table S3). As vertical rainfall (measured through a recording rain gauge) decreased with increased elevation, relative humidity increased with increased elevation. These results highlight the role of relative humidity and leaf wetness acting on the species richness and high individual abundance in high‐elevation areas and species turnover along the gradient. Habitat moisture availability is not accurately captured by rainfall measurements as recorded in rain gauges because the gauges cannot effectively capture horizontal mist and cloud‐produced precipitation (Figure S3). In this study, cloud cover was not directly measured, but relative humidity and leaf wetness were measured and are considered indirect measures of the moisture that cloud cover provides. As elevation increases on Volcán Maderas, cloud cover, relative humidity, leaf wetness, and epiphyte abundance increase.

Similar to other studies in different regions (Cardelús et al., 2006 in Costa Rica, Ding et al., 2016 on Hainan Island, China), our findings suggest that there is a high turnover of vascular epiphyte species on Volcán Maderas and that individual species grow in a narrow elevation range (Tables S1 and S2). Among the five sites sampled on Volcán Maderas, the wet and cloud forest sites had the highest species richness (Figure 2b) and were also most similar in their epiphytic composition (Figure 6). Nonetheless, fewer than half of their species were common to both sites. The clustering analysis shows that the humid forest is more like the wet and cloud forests than it is to the elfin or dry forests. This can be explained by the low prevalence of the Araceae taxa in the elfin forest. In addition, the increased species richness of Piperaceae in the humid forest, separates it from the dry forest, which has very few species of Piperaceae (Figure 3). Interestingly, the Piperaceae species richness remains constant from the humid forest to the cloud forest, and this group contains a single highly diversified genus of epiphytes, *Peperomia*. *Peperomia* is the only genus considered as epizoochorous (having seeds with sticky substances that adhere to animals), thus facilitating long‐distance dispersal (across the elevation gradient). Ferns predominate starting from the wet forest to the elfin forest. Since ferns also require water for sexual reproduction, it is not surprising to find a high diversity of fern species at higher elevations that have increased humidity and leaf wetness and reduced seasonality in moisture.

## 5 CONCLUSION

This study expands our knowledge of elevational patterns for species richness, individual abundance, and turnover of tropical epiphytes by encompassing all vascular epiphyte taxa at sites covering the full extent of the elevational gradient of an isolated, island volcano. We observed (1) a mid‐elevation peak in species richness and increasing abundance, both of which appear to be associated with increasing humidity increasing with elevation (Figure 2a,b); (2) patterns differ across epiphyte taxa (Figure 3), possibly due to different adaptive strategies; and (3) species, for the most part, appear to be narrowly distributed within specific habitat zones along the elevation gradient (Tables S1 and S2).

The narrow elevation bands of epiphyte distribution in these forests make them vulnerable to environmental change, with one of the greatest direct threats being the lifting elevation of the moisture‐laden cloud layer (Beniston et al., 1997; Pouteau et al., 2018; Still et al., 1999), which is often necessary for epiphyte water balance (Darby et al., 2016). Creating realistic estimates for forecasting biotic changes because of a changing climate is crucial for the conservation of tropical montane ecosystems, and this study provides sound baseline data for modeled estimates. Because it is nearly impossible to sample the entire volcano due to steep terrain, timing, and access, our findings are limited to representative data for one side of the mountain elevational gradient. However, these sampled data can be used to create the first solid quantitative extrapolation estimates of species abundance and richness for the volcano with the use of GDM or with machine learning techniques. These approaches will help assess the potential loss of cloud‐forest endemic species impacted by climate change, habitat loss, and biological threats such as invasive species.

## AUTHOR CONTRIBUTIONS

**Hazel K. Berrios:** Conceptualization (equal); data curation (lead); formal analysis (lead); investigation (lead); methodology (lead); project administration (lead); supervision (equal); visualization (lead); writing – original draft (equal); writing – review and editing (equal). **Indiana Coronado:** Project administration (equal); resources (equal); supervision (equal); writing – review and editing (equal). **Travis D. Marsico:** Conceptualization (equal); funding acquisition (lead); investigation (equal); methodology (equal); project administration (equal); supervision (equal); writing – review and editing (equal).

## FUNDING INFORMATION

This work was funded by the Department of Biological Sciences and College of Sciences and Mathematics, Arkansas State University, as well as a faculty research award and an Eleanor Lane Faculty Endowment for International Travel to TDM.

## CONFLICT OF INTEREST

The authors declare no conflict of interest.

### OPEN RESEARCH BADGES

This article has earned an Open Data badge for making publicly available the digitally‐shareable data necessary to reproduce the reported results. The data is available at https://doi.org/10.5061/dryad.bzkh1896h.

## Supporting information


Appendix S1

Appendix S2

## Data Availability

Data are available from the Dryad Digital Repository: https://doi.org/10.5061/dryad.bzkh1896h.
