# Supplementary material for: High species richness and turnover of vascular epiphytes is associated with water availability along the elevation gradient of Volcán Maderas, Nicaragua
Source: Ecol Evol. 2022 Nov 22;12(11):e9501. doi: 10.1002/ece3.9501 (PMC9682193; doi:10.1002/ece3.9501)

**Table S1**

| Family | Forest type | site | Richness | Sampling type |
| --- | --- | --- | --- | --- |
| Araceae | CF | 1060 | 11 | Ss+Os |
| Bromeliaceae | CF | 1060 | 12 | Ss+Os |
| Orchidaceae | CF | 1060 | 24 | Ss+Os |
| Piperaceae | CF | 1060 | 7 | Ss+Os |
| Aspleniaceae | CF | 1060 | 2 | Ss+Os |
| Blechnaceae | CF | 1060 | 2 | Ss+Os |
| Dryopteridaceae | CF | 1060 | 11 | Ss+Os |
| Gleicheniaceae | CF | 1060 | 1 | Ss+Os |
| Hymenophyllaceae | CF | 1060 | 7 | Ss+Os |
| Lomariopsidaceae | CF | 1060 | 0 | Ss+Os |
| Lycopodiaceae | CF | 1060 | 1 | Ss+Os |
| Lygodiaceae | CF | 1060 | 0 | Ss+Os |
| Oleandraceae | CF | 1060 | 1 | Ss+Os |
| Ophioglossaceae | CF | 1060 | 0 | Ss+Os |
| Polypodiaceae | CF | 1060 | 9 | Ss+Os |
| Pteridaceae | CF | 1060 | 0 | Ss+Os |
| Selaginellaceae | CF | 1060 | 2 | Ss+Os |
| Tectariaceae | CF | 1060 | 1 | Ss+Os |
| Thelypteridaceae | CF | 1060 | 1 | Ss+Os |
| Vittariaceae | CF | 1060 | 1 | Ss+Os |
| Begoniaceae | CF | 1060 | 0 | Ss+Os |
| Cactaceae | CF | 1060 | 0 | Ss+Os |
| Clusiaceae | CF | 1060 | 1 | Ss+Os |
| Melastomataceae | CF | 1060 | 0 | Ss+Os |
| Passifloraceae | CF | 1060 | 0 | Ss+Os |
| Araceae | DF | 302 | 7 | Ss+Os |
| Bromeliaceae | DF | 302 | 8 | Ss+Os |
| Orchidaceae | DF | 302 | 10 | Ss+Os |
| Piperaceae | DF | 302 | 1 | Ss+Os |
| Aspleniaceae | DF | 302 | 1 | Ss+Os |
| Blechnaceae | DF | 302 | 0 | Ss+Os |
| Dryopteridaceae | DF | 302 | 1 | Ss+Os |
| Gleicheniaceae | DF | 302 | 0 | Ss+Os |
| Hymenophyllaceae | DF | 302 | 0 | Ss+Os |
| Lomariopsidaceae | DF | 302 | 0 | Ss+Os |
| Lycopodiaceae | DF | 302 | 0 | Ss+Os |
| Lygodiaceae | DF | 302 | 1 | Ss+Os |
| Oleandraceae | DF | 302 | 0 | Ss+Os |
| Ophioglossaceae | DF | 302 | 2 | Ss+Os |
| Polypodiaceae | DF | 302 | 4 | Ss+Os |
| Pteridaceae | DF | 302 | 0 | Ss+Os |
| Selaginellaceae | DF | 302 | 1 | Ss+Os |
| Tectariaceae | DF | 302 | 1 | Ss+Os |
| Thelypteridaceae | DF | 302 | 0 | Ss+Os |
| Vittariaceae | DF | 302 | 0 | Ss+Os |
| Begoniaceae | DF | 302 | 0 | Ss+Os |
| Cactaceae | DF | 302 | 1 | Ss+Os |
| Clusiaceae | DF | 302 | 0 | Ss+Os |
| Melastomataceae | DF | 302 | 0 | Ss+Os |
| Passifloraceae | DF | 302 | 0 | Ss+Os |
| Araceae | EF | 1325 | 2 | Ss+Os |
| Bromeliaceae | EF | 1325 | 3 | Ss+Os |
| Orchidaceae | EF | 1325 | 8 | Ss+Os |
| Piperaceae | EF | 1325 | 3 | Ss+Os |
| Aspleniaceae | EF | 1325 | 0 | Ss+Os |
| Blechnaceae | EF | 1325 | 4 | Ss+Os |
| Dryopteridaceae | EF | 1325 | 7 | Ss+Os |
| Gleicheniaceae | EF | 1325 | 0 | Ss+Os |
| Hymenophyllaceae | EF | 1325 | 6 | Ss+Os |
| Lomariopsidaceae | EF | 1325 | 0 | Ss+Os |
| Lycopodiaceae | EF | 1325 | 2 | Ss+Os |
| Lygodiaceae | EF | 1325 | 0 | Ss+Os |
| Oleandraceae | EF | 1325 | 1 | Ss+Os |
| Ophioglossaceae | EF | 1325 | 0 | Ss+Os |
| Polypodiaceae | EF | 1325 | 10 | Ss+Os |
| Pteridaceae | EF | 1325 | 0 | Ss+Os |
| Selaginellaceae | EF | 1325 | 1 | Ss+Os |
| Tectariaceae | EF | 1325 | 0 | Ss+Os |
| Thelypteridaceae | EF | 1325 | 1 | Ss+Os |
| Vittariaceae | EF | 1325 | 0 | Ss+Os |
| Begoniaceae | EF | 1325 | 0 | Ss+Os |
| Cactaceae | EF | 1325 | 0 | Ss+Os |
| Clusiaceae | EF | 1325 | 0 | Ss+Os |
| Melastomataceae | EF | 1325 | 3 | Ss+Os |
| Passifloraceae | EF | 1325 | 0 | Ss+Os |
| Araceae | HF | 509 | 13 | Ss+Os |
| Bromeliaceae | HF | 509 | 8 | Ss+Os |
| Orchidaceae | HF | 509 | 8 | Ss+Os |
| Piperaceae | HF | 509 | 6 | Ss+Os |
| Aspleniaceae | HF | 509 | 1 | Ss+Os |
| Blechnaceae | HF | 509 | 0 | Ss+Os |
| Dryopteridaceae | HF | 509 | 1 | Ss+Os |
| Gleicheniaceae | HF | 509 | 0 | Ss+Os |
| Hymenophyllaceae | HF | 509 | 0 | Ss+Os |
| Lomariopsidaceae | HF | 509 | 0 | Ss+Os |
| Lycopodiaceae | HF | 509 | 0 | Ss+Os |
| Lygodiaceae | HF | 509 | 0 | Ss+Os |
| Oleandraceae | HF | 509 | 0 | Ss+Os |
| Ophioglossaceae | HF | 509 | 0 | Ss+Os |
| Polypodiaceae | HF | 509 | 6 | Ss+Os |
| Pteridaceae | HF | 509 | 1 | Ss+Os |
| Selaginellaceae | HF | 509 | 2 | Ss+Os |
| Tectariaceae | HF | 509 | 0 | Ss+Os |
| Thelypteridaceae | HF | 509 | 0 | Ss+Os |
| Vittariaceae | HF | 509 | 0 | Ss+Os |
| Begoniaceae | HF | 509 | 1 | Ss+Os |
| Cactaceae | HF | 509 | 0 | Ss+Os |
| Clusiaceae | HF | 509 | 1 | Ss+Os |
| Melastomataceae | HF | 509 | 0 | Ss+Os |
| Passifloraceae | HF | 509 | 0 | Ss+Os |
| Araceae | WF | 890 | 13 | Ss+Os |
| Bromeliaceae | WF | 890 | 12 | Ss+Os |
| Orchidaceae | WF | 890 | 24 | Ss+Os |
| Piperaceae | WF | 890 | 7 | Ss+Os |
| Aspleniaceae | WF | 890 | 1 | Ss+Os |
| Blechnaceae | WF | 890 | 1 | Ss+Os |
| Dryopteridaceae | WF | 890 | 5 | Ss+Os |
| Gleicheniaceae | WF | 890 | 0 | Ss+Os |
| Hymenophyllaceae | WF | 890 | 3 | Ss+Os |
| Lomariopsidaceae | WF | 890 | 1 | Ss+Os |
| Lycopodiaceae | WF | 890 | 0 | Ss+Os |
| Lygodiaceae | WF | 890 | 0 | Ss+Os |
| Oleandraceae | WF | 890 | 1 | Ss+Os |
| Ophioglossaceae | WF | 890 | 0 | Ss+Os |
| Polypodiaceae | WF | 890 | 5 | Ss+Os |
| Pteridaceae | WF | 890 | 2 | Ss+Os |
| Selaginellaceae | WF | 890 | 4 | Ss+Os |
| Tectariaceae | WF | 890 | 1 | Ss+Os |
| Thelypteridaceae | WF | 890 | 0 | Ss+Os |
| Vittariaceae | WF | 890 | 0 | Ss+Os |
| Begoniaceae | WF | 890 | 0 | Ss+Os |
| Cactaceae | WF | 890 | 0 | Ss+Os |
| Clusiaceae | WF | 890 | 1 | Ss+Os |
| Melastomataceae | WF | 890 | 0 | Ss+Os |
| Passifloraceae | WF | 890 | 1 | Ss+Os |
| Araceae | DF | 302 | 7 | Ss |
| Araceae | HF | 509 | 11 | Ss |
| Araceae | WF | 890 | 12 | Ss |
| Araceae | CF | 1060 | 11 | Ss |
| Araceae | EF | 1325 | 2 | Ss |
| Bromeliaceae | DF | 302 | 6 | Ss |
| Bromeliaceae | HF | 509 | 3 | Ss |
| Bromeliaceae | WF | 890 | 8 | Ss |
| Bromeliaceae | CF | 1060 | 8 | Ss |
| Bromeliaceae | EF | 1325 | 2 | Ss |
| Orchidaceae | DF | 302 | 9 | Ss |
| Orchidaceae | HF | 509 | 7 | Ss |
| Orchidaceae | WF | 890 | 24 | Ss |
| Orchidaceae | CF | 1060 | 21 | Ss |
| Orchidaceae | EF | 1325 | 8 | Ss |
| Piperaceae | DF | 302 | 1 | Ss |
| Piperaceae | HF | 509 | 6 | Ss |
| Piperaceae | WF | 890 | 7 | Ss |
| Piperaceae | CF | 1060 | 7 | Ss |
| Piperaceae | EF | 1325 | 3 | Ss |
| Aspleniaceae | DF | 302 | 1 | Ss |
| Aspleniaceae | HF | 509 | 1 | Ss |
| Aspleniaceae | WF | 890 | 0 | Ss |
| Aspleniaceae | CF | 1060 | 0 | Ss |
| Aspleniaceae | EF | 1325 | 0 | Ss |
| Blechnaceae | DF | 302 | 0 | Ss |
| Blechnaceae | HF | 509 | 0 | Ss |
| Blechnaceae | WF | 890 | 0 | Ss |
| Blechnaceae | CF | 1060 | 0 | Ss |
| Blechnaceae | EF | 1325 | 1 | Ss |
| Dryopteridaceae | DF | 302 | 1 | Ss |
| Dryopteridaceae | HF | 509 | 1 | Ss |
| Dryopteridaceae | WF | 890 | 3 | Ss |
| Dryopteridaceae | CF | 1060 | 8 | Ss |
| Dryopteridaceae | EF | 1325 | 5 | Ss |
| Gleicheniaceae | DF | 302 | 0 | Ss |
| Gleicheniaceae | HF | 509 | 0 | Ss |
| Gleicheniaceae | WF | 890 | 0 | Ss |
| Gleicheniaceae | CF | 1060 | 1 | Ss |
| Gleicheniaceae | EF | 1325 | 0 | Ss |
| Hymenophyllaceae | DF | 302 | 0 | Ss |
| Hymenophyllaceae | HF | 509 | 0 | Ss |
| Hymenophyllaceae | WF | 890 | 2 | Ss |
| Hymenophyllaceae | CF | 1060 | 5 | Ss |
| Hymenophyllaceae | EF | 1325 | 5 | Ss |
| Lycopodiaceae | DF | 302 | 0 | Ss |
| Lycopodiaceae | HF | 509 | 0 | Ss |
| Lycopodiaceae | WF | 890 | 0 | Ss |
| Lycopodiaceae | CF | 1060 | 1 | Ss |
| Lycopodiaceae | EF | 1325 | 2 | Ss |
| Oleandraceae | DF | 302 | 0 | Ss |
| Oleandraceae | HF | 509 | 0 | Ss |
| Oleandraceae | WF | 890 | 1 | Ss |
| Oleandraceae | CF | 1060 | 1 | Ss |
| Oleandraceae | EF | 1325 | 1 | Ss |
| Ophioglossaceae | DF | 302 | 2 | Ss |
| Ophioglossaceae | HF | 509 | 0 | Ss |
| Ophioglossaceae | WF | 890 | 0 | Ss |
| Ophioglossaceae | CF | 1060 | 0 | Ss |
| Ophioglossaceae | EF | 1325 | 0 | Ss |
| Polypodiaceae | DF | 302 | 4 | Ss |
| Polypodiaceae | HF | 509 | 2 | Ss |
| Polypodiaceae | WF | 890 | 1 | Ss |
| Polypodiaceae | CF | 1060 | 5 | Ss |
| Polypodiaceae | EF | 1325 | 4 | Ss |
| Pteridaceae | DF | 302 | 0 | Ss |
| Pteridaceae | HF | 509 | 0 | Ss |
| Pteridaceae | WF | 890 | 2 | Ss |
| Pteridaceae | CF | 1060 | 0 | Ss |
| Pteridaceae | EF | 1325 | 0 | Ss |
| Selaginellaceae | DF | 302 | 1 | Ss |
| Selaginellaceae | HF | 509 | 2 | Ss |
| Selaginellaceae | WF | 890 | 2 | Ss |
| Selaginellaceae | CF | 1060 | 2 | Ss |
| Selaginellaceae | EF | 1325 | 1 | Ss |
| Tectariaceae | DF | 302 | 1 | Ss |
| Tectariaceae | HF | 509 | 0 | Ss |
| Tectariaceae | WF | 890 | 1 | Ss |
| Tectariaceae | CF | 1060 | 0 | Ss |
| Tectariaceae | EF | 1325 | 0 | Ss |
| Thelypteridaceae | DF | 302 | 0 | Ss |
| Thelypteridaceae | HF | 509 | 0 | Ss |
| Thelypteridaceae | WF | 890 | 0 | Ss |
| Thelypteridaceae | CF | 1060 | 1 | Ss |
| Thelypteridaceae | EF | 1325 | 0 | Ss |
| Cactaceae | DF | 302 | 1 | Ss |
| Cactaceae | HF | 509 | 0 | Ss |
| Cactaceae | WF | 890 | 0 | Ss |
| Cactaceae | CF | 1060 | 0 | Ss |
| Cactaceae | EF | 1325 | 0 | Ss |
| Clusiaceae | DF | 302 | 0 | Ss |
| Clusiaceae | HF | 509 | 1 | Ss |
| Clusiaceae | WF | 890 | 1 | Ss |
| Clusiaceae | CF | 1060 | 1 | Ss |
| Clusiaceae | EF | 1325 | 0 | Ss |
| Melastomataceae | DF | 302 | 0 | Ss |
| Melastomataceae | HF | 509 | 0 | Ss |
| Melastomataceae | WF | 890 | 0 | Ss |
| Melastomataceae | CF | 1060 | 0 | Ss |
| Melastomataceae | EF | 1325 | 2 | Ss |
| Passifloraceae | DF | 302 | 0 | Ss |
| Passifloraceae | HF | 509 | 0 | Ss |
| Passifloraceae | WF | 890 | 1 | Ss |
| Passifloraceae | CF | 1060 | 0 | Ss |
| Passifloraceae | EF | 1325 | 0 | Ss |
| Araceae | DF | 302 | 0 | OS |
| Araceae | HF | 509 | 2 | OS |
| Araceae | WF | 890 | 1 | OS |
| Araceae | CF | 1060 | 0 | OS |
| Araceae | EF | 1325 | 0 | OS |
| Bromeliaceae | DF | 302 | 2 | OS |
| Bromeliaceae | HF | 509 | 5 | OS |
| Bromeliaceae | WF | 890 | 4 | OS |
| Bromeliaceae | CF | 1060 | 4 | OS |
| Bromeliaceae | EF | 1325 | 1 | OS |
| Orchidaceae | DF | 302 | 1 | OS |
| Orchidaceae | HF | 509 | 1 | OS |
| Orchidaceae | WF | 890 | 0 | OS |
| Orchidaceae | CF | 1060 | 3 | OS |
| Orchidaceae | EF | 1325 | 0 | OS |
| Piperaceae | DF | 302 | 0 | OS |
| Piperaceae | HF | 509 | 0 | OS |
| Piperaceae | WF | 890 | 0 | OS |
| Piperaceae | CF | 1060 | 0 | OS |
| Piperaceae | EF | 1325 | 0 | OS |
| Aspleniaceae | DF | 302 | 0 | OS |
| Aspleniaceae | HF | 509 | 0 | OS |
| Aspleniaceae | WF | 890 | 1 | OS |
| Aspleniaceae | CF | 1060 | 2 | OS |
| Aspleniaceae | EF | 1325 | 0 | OS |
| Blechnaceae | DF | 302 | 0 | OS |
| Blechnaceae | HF | 509 | 0 | OS |
| Blechnaceae | WF | 890 | 1 | OS |
| Blechnaceae | CF | 1060 | 2 | OS |
| Blechnaceae | EF | 1325 | 3 | OS |
| Dryopteridaceae | DF | 302 | 0 | OS |
| Dryopteridaceae | HF | 509 | 0 | OS |
| Dryopteridaceae | WF | 890 | 2 | OS |
| Dryopteridaceae | CF | 1060 | 3 | OS |
| Dryopteridaceae | EF | 1325 | 2 | OS |
| Gleicheniaceae | DF | 302 | 0 | OS |
| Gleicheniaceae | HF | 509 | 0 | OS |
| Gleicheniaceae | WF | 890 | 0 | OS |
| Gleicheniaceae | CF | 1060 | 0 | OS |
| Gleicheniaceae | EF | 1325 | 0 | OS |
| Hymenophyllaceae | DF | 302 | 0 | OS |
| Hymenophyllaceae | HF | 509 | 0 | OS |
| Hymenophyllaceae | WF | 890 | 1 | OS |
| Hymenophyllaceae | CF | 1060 | 2 | OS |
| Hymenophyllaceae | EF | 1325 | 1 | OS |
| Lomariopsidaceae | DF | 302 | 0 | OS |
| Lomariopsidaceae | HF | 509 | 0 | OS |
| Lomariopsidaceae | WF | 890 | 1 | OS |
| Lomariopsidaceae | CF | 1060 | 0 | OS |
| Lomariopsidaceae | EF | 1325 | 0 | OS |
| Lycopodiaceae | DF | 302 | 0 | OS |
| Lycopodiaceae | HF | 509 | 0 | OS |
| Lycopodiaceae | WF | 890 | 0 | OS |
| Lycopodiaceae | CF | 1060 | 0 | OS |
| Lycopodiaceae | EF | 1325 | 0 | OS |
| Lygodiaceae | DF | 302 | 1 | OS |
| Lygodiaceae | HF | 509 | 0 | OS |
| Lygodiaceae | WF | 890 | 0 | OS |
| Lygodiaceae | CF | 1060 | 0 | OS |
| Lygodiaceae | EF | 1325 | 0 | OS |
| Oleandraceae | DF | 302 | 0 | OS |
| Oleandraceae | HF | 509 | 0 | OS |
| Oleandraceae | WF | 890 | 0 | OS |
| Oleandraceae | CF | 1060 | 0 | OS |
| Oleandraceae | EF | 1325 | 0 | OS |
| Ophioglossaceae | DF | 302 | 0 | OS |
| Ophioglossaceae | HF | 509 | 0 | OS |
| Ophioglossaceae | WF | 890 | 0 | OS |
| Ophioglossaceae | CF | 1060 | 0 | OS |
| Ophioglossaceae | EF | 1325 | 0 | OS |
| Polypodiaceae | DF | 302 | 0 | OS |
| Polypodiaceae | HF | 509 | 4 | OS |
| Polypodiaceae | WF | 890 | 4 | OS |
| Polypodiaceae | CF | 1060 | 4 | OS |
| Polypodiaceae | EF | 1325 | 6 | OS |
| Pteridaceae | DF | 302 | 0 | OS |
| Pteridaceae | HF | 509 | 1 | OS |
| Pteridaceae | WF | 890 | 0 | OS |
| Pteridaceae | CF | 1060 | 0 | OS |
| Pteridaceae | EF | 1325 | 0 | OS |
| Selaginellaceae | DF | 302 | 0 | OS |
| Selaginellaceae | HF | 509 | 0 | OS |
| Selaginellaceae | WF | 890 | 2 | OS |
| Selaginellaceae | CF | 1060 | 0 | OS |
| Selaginellaceae | EF | 1325 | 0 | OS |
| Tectariaceae | DF | 302 | 0 | OS |
| Tectariaceae | HF | 509 | 0 | OS |
| Tectariaceae | WF | 890 | 0 | OS |
| Tectariaceae | CF | 1060 | 1 | OS |
| Tectariaceae | EF | 1325 | 0 | OS |
| Thelypteridaceae | DF | 302 | 0 | OS |
| Thelypteridaceae | HF | 509 | 0 | OS |
| Thelypteridaceae | WF | 890 | 0 | OS |
| Thelypteridaceae | CF | 1060 | 0 | OS |
| Thelypteridaceae | EF | 1325 | 1 | OS |
| Vittariaceae | DF | 302 | 0 | OS |
| Vittariaceae | HF | 509 | 0 | OS |
| Vittariaceae | WF | 890 | 0 | OS |
| Vittariaceae | CF | 1060 | 1 | OS |
| Vittariaceae | EF | 1325 | 0 | OS |
| Begoniaceae | DF | 302 | 0 | OS |
| Begoniaceae | HF | 509 | 1 | OS |
| Begoniaceae | WF | 890 | 0 | OS |
| Begoniaceae | CF | 1060 | 0 | OS |
| Begoniaceae | EF | 1325 | 0 | OS |
| Cactaceae | DF | 302 | 0 | OS |
| Cactaceae | HF | 509 | 0 | OS |
| Cactaceae | WF | 890 | 0 | OS |
| Cactaceae | CF | 1060 | 0 | OS |
| Cactaceae | EF | 1325 | 0 | OS |
| Clusiaceae | DF | 302 | 0 | OS |
| Clusiaceae | HF | 509 | 0 | OS |
| Clusiaceae | WF | 890 | 0 | OS |
| Clusiaceae | CF | 1060 | 0 | OS |
| Clusiaceae | EF | 1325 | 0 | OS |
| Melastomataceae | DF | 302 | 0 | OS |
| Melastomataceae | HF | 509 | 0 | OS |
| Melastomataceae | WF | 890 | 0 | OS |
| Melastomataceae | CF | 1060 | 0 | OS |
| Melastomataceae | EF | 1325 | 1 | OS |
| Passifloraceae | DF | 302 | 0 | OS |
| Passifloraceae | HF | 509 | 0 | OS |
| Passifloraceae | WF | 890 | 0 | OS |
| Passifloraceae | CF | 1060 | 0 | OS |
| Passifloraceae | EF | 1325 | 0 | OS |

**Table S2**

| **Family** | **Species** | **Forest Type** | | | | |
| --- | --- | --- | --- | --- | --- | --- |
|  |  | **DF** | **HF** | **WF** | **CF** | **EF** |
| **Araceae** | *Anthurium bakeri* Hook. f. | **̶** | **○** | **◊** | **○** | **̶** |
|  | *Anthurium cubense* Engl. | **○** | **̶** | **̶** | **̶** | **̶** |
|  | *Anthurium flexile* Schott | **̶** | ***** | **○** | **◊** | **̶** |
|  | *Anthurium scandens* (Aubl.) Engl. in Mart. | **◊** | ***** | **○** | **̶** | **̶** |
|  | Anthurium sp.1 | **◊** | **̶** | **̶** | **◊** | **̶** |
|  | Araceae unknown 1 | **̶** | **̶** | **̶** | **◊** | **̶** |
|  | Araceae unknown 2 | **̶** | **̶** | **̶** | **◊** | **̶** |
|  | Araceae unknown 3 | **̶** | **◊** | **̶** | **̶** | **̶** |
|  | Araceae unknown 4 | **̶** | **̶** | **◊** | **◊** | **̶** |
|  | Araceae unknown 5 | **̶** | **̶** | **̶** | **◊** | **̶** |
|  | Araceae unknown 6 | **̶** | **̶** | **̶** | **◊** | **̶** |
|  | Araceae unknown 7 | **̶** | **̶** | **◊** | **̶** | **̶** |
|  | Araceae unknown 8 | **◊** | **̶** | **̶** | **̶** | **̶** |
|  | *Monstera adansonii* Schott var. *laniata* (Schott) Madison | **○** | **○** | **○** | **◊** | **○** |
|  | *Philodendron aurantiifolium* Schott subsp *.calderense* (K. Krause) Grayum | **̶** | **○** | **◊** | **◊** | **◊** |
|  | *Philodendron hederaceum* (Jacq.) Schott | **◊** | **○** | **◊** | **◊** | **̶** |
|  | *Philodendron tenue* K. Koch & Augustin | **̶** | **◊** | **○** | **○** | **̶** |
|  | *Syngonium hoffmannii* Schott | **̶** | **○** | **◊** | **̶** | **̶** |
|  | *Syngonium podophyllum* Schott | **○** | ***** | **◊** | **̶** | **̶** |
|  | *Syngonium* sp. 1 | **̶** | **◊** |  | **̶** | **̶** |
| **Aspleniaceae** | *Asplenium miradorense* Liebm. | **̶** | **̶** | **̶** | ***** | **̶** |
|  | *Asplenium pteropus* Kaulf. | **◊** | ***** | **̶** | **̶** | **̶** |
|  | *Asplenium serra* Langsd. & Fisch. | **̶** | **̶** | ***** | **̶** | **̶** |
| **Begoniaceae** | *Begonia glabra* Aubl. | **̶** | ***** | **̶** | **̶** | **̶** |
| **Blechnaceae** | *Blechnum fragile* (Liebm.) C.V. Morton & Lellinger | **̶** | **̶** | **̶** | **̶** | **○** |
|  | *Salpichlaena volubilis* (Kaulf.) J. Sm. | **̶** | **̶** | ***** | **̶** | ***** |
| **Bromeliaceae** | *Aechmea bracteata* (Sw.) Griseb. | ***** | **̶** | **̶** | **̶** | **̶** |
|  | Bromeliaceae unknown 1 | **̶** | **̶** | **̶** | **◊** | **̶** |
|  | Bromeliaceae unknown 2 | **̶** | **̶** | **◊** | **̶** | **̶** |
|  | Bromeliaceae unknown 3 | **̶** | **̶** | **̶** | **◊** | **̶** |
|  | Bromeliaceae unknown 4 | **◊** | **̶** | **̶** | **̶** | **̶** |
|  | Bromeliaceae unknown 5 | **̶** | **̶** | **◊** | **̶** | **̶** |
|  | *Catopsis morreniana* Mez | **̶** | **̶** | ***** | **̶** | **̶** |
|  | *Guzmania angustifolia* (Baker) Wittm. | **̶** | **̶** | **̶** | ***** | **̶** |
|  | *Guzmania lingulata* (L.) Mez in C. DC. | **̶** | **̶** | **̶** | **○** | **◊** |
|  | *Guzmania monostachia* (L.) Rusby ex Mez in C. DC. | **̶** | ***** | **̶** | **̶** | **̶** |
|  | *Guzmania nicaraguensis* Mez & C.F. Baker | **̶** | **̶** | **̶** | **○** | **̶** |
|  | *Guzmania* sp. 1 | **̶** | **̶** | **̶** | **◊** | **̶** |
|  | *Pitcairnia imbricata* (Brongn.) Regel | **̶** | **̶** | ***** | ***** | **̶** |
|  | *Tilandsia anceps* G. Lodd | **̶** | **̶** | **◊** | **̶** | **̶** |
|  | *Tillandsia bulbosa* Hook. | **̶** | ***** | **̶** | **̶** | **̶** |
|  | *Tillandsia fasciculata* Sw. | **◊** | **̶** | **̶** | **̶** | **̶** |
|  | *Tillandsia juncea* (Ruiz & Pav.) Poir. | **̶** | ***** | **̶** | **̶** | **̶** |
|  | *Tillandsia leiboldiana* Schltdl. | **̶** | **̶** | **̶** | ***** | **̶** |
|  | *Tillandsia monadelpha* (E. Morren) Baker | **◊** | ***** | ***** | **̶** | **̶** |
|  | *Tilandsia paucifolia* Baker | **̶** | **̶** | **◊** | **̶** | **̶** |
|  | *Tilandsia* sp.1 | **̶** | **◊** | **◊** | **◊** | **̶** |
|  | *Tillandsia schiedeana* Steud. | **○** | **○** | **̶** | **̶** | **̶** |
|  | *Tillandsia tricolor* Schltdl. & Cham. | ***** | **̶** | **̶** | **̶** | **̶** |
|  | *Tillandsia usneoides* (L.) L. | **̶** | ***** | ***** | **̶** | **̶** |
|  | *Vriesea pedicellata* (Mez & Wercklé) L.B. Sm. & Pittendr. | **̶** | **̶** | **̶** | ***** | ***** |
|  | *Vriesea sanguinolenta* Cogn. & Marchal | **◊** | ***** | **◊** | **̶** | **◊** |
|  | *Vriesea* sp.1 | **◊** | **̶** | **◊** | **◊** | **̶** |
|  | *Vriesea viridiflora* (Regel) Wittm. ex Mez | **̶** | **̶** | **◊** | **̶** | **̶** |
|  | *Vriesea vittata* (Mez & Wercklé) L.B. Sm. & Pittendr. | **̶** | **̶** | **̶** | ***** | **̶** |
| **Cactaceae** | *Hylocereus costaricensis* (F.A.C. Weber) Britton & Rose | **◊** | **̶** | **̶** | **̶** | **̶** |
| **Clusiaceae** | Clusiaceae sp. 1 | **̶** | **◊** | **◊** |  | **̶** |
| **Dryopteridaceae** | *Ctenitis melanosticta* (Kunze) Copel. | **̶** | **̶** | **̶** | **◊** | **̶** |
|  | *Elaphoglossum crinitum* (L.) H. Christ | **̶** | **̶** | ***** | ***** | **̶** |
|  | *Elaphoglossum erinaceum* (Fee) T. Moore var. *erinaceum* | **̶** | **̶** | **̶** | **̶** | **◊** |
|  | *Elaphoglossum eximium* (Mett.) H. Christ | **̶** | **̶** | **̶** | **◊** | **̶** |
|  | *Elaphoglossum furfuraceum* (Mett. Ex Kuhn) H. Christ | **̶** | **̶** | **̶** | **◊** | **◊** |
|  | *Elaphoglossum nigrescens* (Hook.) T. Moore ex Diels en Engl. | **̶** | **̶** | **○** | **○** | **̶** |
|  | *Elaphoglossum peltatum* (Sw.) Urb. | **̶** | **̶** | **◊** | **○** | **̶** |
|  | *Elaphoglossum setosum* (Liebm.) T. Moore | **̶** | **̶** | **̶** | ***** | ***** |
|  | *Elaphoglossum* sp. 1 | **◊** | **◊** | **◊** | **◊** |  |
|  | *Elaphoglossum* sp. 2 | **̶** | **̶** | **̶** | **◊** | **̶** |
|  | *Elaphoglossum* sp. 3 | **̶** | **̶** | **̶** | **̶** | **◊** |
|  | *Elaphoglossum* sp. 4 | **̶** | **̶** | **̶** | **◊** | **̶** |
|  | *Elaphoglossum* sp. 5 | **̶** | **̶** | **̶** | **̶** | **○** |
|  | *Polybotrya alfredii* Brade | **̶** | **̶** | ***** | **̶** | **̶** |
| **Gleicheniaceae** | *Sticherus fulvus* (Desv.) Ching | **̶** | **̶** | **̶** | **◊** | **̶** |
| **Hymenophyllaceae** | *Hymenophyllum asplenioides* (Sw.) Sw. | **̶** | **̶** | **̶** | **○** | **◊** |
|  | *Hymenophyllum consanguineum* C.V. Morton | **̶** | **̶** | **̶** | **̶** | **○** |
|  | *Hymenophyllum polyanthos* (Sw.) Sw. | **̶** | **̶** | **̶** | **○** | **◊** |
|  | Hymenophyllum sp.1 | **̶** | **̶** | **◊** | **◊** | **̶** |
|  | *Trichomanes collariatum* Bosch | **̶** | **̶** | ***** | **̶** | **̶** |
|  | *Trichomanes galeottii* E. Fourn. | **̶** | **̶** | **○** | ***** | ***** |
|  | *Trichomanes radicans* Sw. | **̶** | **̶** | **̶** | ***** | **̶** |
|  | *Trichomanes* sp. 1 | **̶** | **̶** | **̶** | **◊** | **◊** |
| **Lomariopsidaceae** | *Lomariopsis vestita* E. Fourn. | **̶** | **̶** | ***** | **̶** | **̶** |
| **Lycopodiaceae** | *Huperzia linifolia* (L.) Trevis. | **̶** | **̶** | **̶** | ***** | **◊** |
|  | *Lycopodiella cernua* (L.) Pic. Sern | **̶** | **̶** | **̶** | **̶** | **◊** |
| **Lygodiaceae** | *Lygodium venustum* Sw. | ***** | **̶** | **̶** | **̶** | **̶** |
| **Melastomataceae** | *Conostegia* sp. 1 | **̶** | **̶** | **̶** | **̶** | **◊** |
|  | *Conostegia* sp. 2 | **̶** | **̶** | **̶** | **̶** | **◊** |
| **Oleandraceae** | *Oleandra articulata* (Sw.) C. Presl | **̶** | **̶** | **◊** | **◊** | **○** |
| **Ophioglossaceae** | *Ophioglossum palmatum* L. | **◊** | **̶** | **̶** | **̶** | **̶** |
|  | *Ophioglossum reticulatum* L. | **◊** | **̶** | **̶** | **̶** | **̶** |
| **Orchidaceae** | *Elleanthus wercklei* Schltr. | **̶** | **̶** | **̶** | ***** | **̶** |
|  | *Epidendrum cardiochilum* L.O. Williams | **̶** | **̶** | **◊** | **̶** | **̶** |
|  | *Epidendrum Phragmites* A.H. Heller & L.O. Williams | **̶** | **̶** | **◊** | **̶** | **̶** |
|  | *Epidendrum ramosum* Jacq. | **◊** | **◊** | **̶** | **̶** | **̶** |
|  | *Epidendrum* sp*.*1 | **◊** |  | **◊** | **◊** |  |
|  | *Epidendrum* sp. 2 | **̶** | **̶** | **̶** | **̶** | **◊** |
|  | *Epidendrum* sp. 3 | **̶** | **̶** | **̶** | **̶** | **◊** |
|  | *Gongora claviodora* Dressler | **◊** | ***** | **̶** | **̶** | **̶** |
|  | *Jacquiniella globosa* (Jacq.) Schltr. | **̶** | **̶** | **◊** | **◊** | **̶** |
|  | *Lepanthes blepharistes* Rchb. f. | **̶** | **̶** | **◊** | **○** |  |
|  | *Lepanthes* sp. 1 | **̶** | **̶** | **◊** | **̶** | **̶** |
|  | *Lycaste cochleata* Lindl. | **̶** | **̶** | **̶** | **◊** | **̶** |
|  | *Masdevallia chontalensis* Rchb. f. | **̶** | **̶** | **̶** | ***** | **̶** |
|  | *Maxillaria friedrichsthallii* Rchb. f. | ***** | ***** | **̶** | **̶** | **̶** |
|  | *Maxillaria mombachoensis* A.H. Heller ex J.T. Atwood | **̶** | **̶** | ***** | **○** | **̶** |
|  | *Myrmecophila* sp. 1 | **̶** | **̶** | **̶** | **◊** | **̶** |
|  | *Myrmecophila tibicinis* (Bateman) Rolfe | **◊** | **̶** | **̶** | **̶** | **̶** |
|  | Orchidaceae unknown 1 | **̶** | **̶** | **◊** | **̶** | **̶** |
|  | Orchidaceae unknown 2 | **̶** | **̶** | **◊** | **◊** | **̶** |
|  | Orchidaceae unknown 3 | **̶** | **̶** | **◊** | **̶** | **̶** |
|  | Orchidaceae unknown 4 | **̶** | **̶** | **̶** | **◊** | **̶** |
|  | Orchidaceae unknown 5 | **̶** | **̶** | **◊** | **̶** | **̶** |
|  | Orchidaceae unknown 6 | **̶** | **̶** | **◊** | **̶** | **̶** |
|  | Orchidaceae unknown 7 | **̶** | **̶** | **̶** | **◊** | **̶** |
|  | Orchidaceae unknown 8 | **◊** | **̶** | **̶** | **̶** | **̶** |
|  | Orchidaceae unknown 9 | **̶** | **̶** | **̶** | **̶** | **◊** |
|  | Orchidaceae unknown 10 | **̶** | **̶** | **◊** | **̶** | **̶** |
|  | Orchidaceae unknown 11 | **̶** | **◊** | **̶** | **̶** | **̶** |
|  | Orchidaceae unknown 12 | **̶** | **̶** | **◊** | **̶** | **̶** |
|  | Orchidaceae unknown 13 | **̶** | **̶** | **̶** | **̶** | **◊** |
|  | Orchidaceae unknown 14 | **̶** | **̶** | **̶** | **◊** | **̶** |
|  | Orchidaceae unknown 15 | **◊** | **̶** | **̶** | **̶** | **̶** |
|  | Orchidaceae unknown 16 | **̶** | **̶** | **̶** | **◊** | **̶** |
|  | Orchidaceae unknown 17 | **̶** | **̶** | **◊** | **̶** | **̶** |
|  | Orchidaceae unknown 18 | **̶** | **̶** | **◊** | **̶** | **̶** |
|  | *Platystele compacta* (Ames) Ames | **̶** | **̶** | **◊** | **̶** | **̶** |
|  | *Platystele* sp. 1 | **̶** | **̶** | **̶** | **̶** | **◊** |
|  | *Pleurothallis* sp*.* 1 | **̶** | **◊** | **◊** | **◊** | **̶** |
|  | *Pleurothallis uncinata* Fawc. | **̶** | **̶** | **̶** | **◊** | **̶** |
|  | *Ponthieva* sp. 1 | **̶** | **̶** | **̶** | **◊** | **̶** |
|  | *Prosthechea cochleata* (L.) W.E. Higgins | **◊** | **̶** | **̶** | **◊** | **◊** |
|  | *Restrepiella ophiocephala* (Lindl.) Garay & Dunst. | **̶** | **̶** | **̶** | **◊** | **̶** |
|  | *Scaphyglottis micrantha* (Lindl.) Ames & Correll | **○** | **̶** | **̶** | **̶** | **̶** |
|  | *Scaphyglottis prolifera* (Sw.) Cogn. | **̶** | ***** | **○** | **̶** | **̶** |
|  | *Scaphyglottis* sp.1 | **̶** | **◊** | **◊** | **◊** | **̶** |
|  | *Sigmatostalix* sp. 1 | **̶** | **◊** | **̶** | **̶** | **̶** |
|  | *Sobralia decora* Bateman | **̶** | **̶** | **̶** | ***** | **̶** |
|  | *Sobralia* sp.1 | **̶** | **̶** | **̶** | **◊** | **◊** |
|  | *Stelis argentata* Lindl | **̶** | **̶** | **◊** | **̶** | **̶** |
|  | *Stelis gracilis* Ames | **̶** | **̶** | **○** | **◊** | **◊** |
|  | *Stelis* sp.1 | **◊** | **̶** | **◊** | **◊** | **̶** |
|  | *Trichosalpinx dura* (Lindl.) Luer | **̶** | **̶** | **̶** | **◊** | **̶** |
|  | *Vanilla pompona* Schiede | **̶** | **̶** | **◊** | **̶** | **̶** |
|  | *Vanilla* sp. 1 | **̶** | **̶** | **̶** | **̶** | **◊** |
| **Piperaceae** | *Peperomia cyclophylla* Miq. | **̶** | **◊** | **̶** | **̶** | **̶** |
|  | *Peperomia deppeana* Schltdl. & Cham. | **̶** | **○** | **̶** | ***** | **̶** |
|  | *Peperomia dotana* Trel. | **̶** | **◊** | **◊** | **◊** | **○** |
|  | *Peperomia emarginella* (Sw. ex Wikstr.) C. DC. | **◊** | **̶** | ***** | **̶** | **̶** |
|  | *Peperomia glabella* (Sw.) A. Dietr. | **̶** | ***** | **○** | **◊** | **̶** |
|  | *Peperomia obtusifolia* (L.) A. Dietr. | **̶** | **̶** | **○** | **○** | **◊** |
|  | *Peperomia portobellensis* Beurl. | **̶** | **○** | **̶** | **̶** | **̶** |
|  | *Peperomia* sp. 1 | **̶** | **◊** | **◊** | **◊** | **◊** |
|  | *Peperomia urocarpa* Fisch. & C.A. Mey. | **̶** | **̶** | **○** | ***** | **̶** |
|  | Piperaceae unknown 1 | **̶** | **̶** | **̶** | **◊** | **̶** |
|  | Piperaceae unknown 2 | **̶** | **̶** | **◊** | **̶** | **̶** |
| **Passifloraceae** | Passifloraceae unknown 1 | **̶** | **̶** | **◊** | **̶** | **̶** |
| **Polypodiaceae** | *Campyloneurum brevifolium* (Lodd. ex Link) Link | **̶** | ***** | **̶** | **̶** | **̶** |
|  | *Campyloneurum repens* (Aubl.) C. Presl | **̶** | **̶** | ***** | **̶** | **̶** |
|  | *Cochlidium rostratum* (Hook.) Maxon ex C. Chr. | **̶** | **̶** | **̶** | ***** | **○** |
|  | *Cochlidium serrulatum* (Sw.) L.E. Bishop | **̶** | **̶** | **̶** | **̶** | **○** |
|  | *Lellingeria melanotrichia* A.R. Sm. & R.C. Moran | **̶** | **̶** | **̶** | **̶** | **○** |
|  | *Lellingeria mitchelliae* (Baker) A.R. Sm. & R.C. Moran | **̶** | **̶** | **○** | **◊** | **◊** |
|  | *Lellingeria prionodes* (Mickel & Beitel) A.R. Sm. & R.C. Moran | **̶** | **̶** | **̶** | **̶** | ***** |
|  | *Lellingeria suspensa* (L.) A.R. Sm. & R.C. Moran | **̶** | **̶** | **̶** | **̶** | ***** |
|  | *Microgramma lycopodioides* (L.) Copel. | **̶** | ***** | **̶** | **̶** | **̶** |
|  | *Microgramma percussa* (Cav.) de la Sota | **̶** | ***** | **̶** | **̶** | **̶** |
|  | *Microgramma reptans* (Cav.) A.R. Sm | **̶** | **̶** | **̶** | **̶** | **◊** |
|  | *Micropolypodium taenifolium* (Jenman) A.R. Sm. | **̶** | **̶** | ***** | ***** | ***** |
|  | *Pecluma plumula* (Humb. & Bonpl. ex Willd.) M.G. Price | **̶** | **̶** | ***** | **̶** | **̶** |
|  | *Phlebodium pseudoaureum* (Cav.) Lellinger | **◊** | **̶** | **̶** | **̶** | **̶** |
|  | *Pleopeltis astrolepis* (Liebm.) E. Fourn. | **̶** | ***** | **̶** | **̶** | **̶** |
|  | *Pleopeltis macrocarpa* (Bory ex Willd.) Kaulf. var. macrocarpa |  | **◊** | **̶** | ***** | **̶** |
|  | *Pleopeltis polypodioides* (L.) E.G. Andrews & Windham | **◊** | **̶** | **̶** | **◊** | **̶** |
|  | *Polypodium dulce* Poir | **̶** | **̶** | **̶** | **◊** | **̶** |
|  | *Polypodium plesiosorum* Kunze | **̶** | **̶** | **̶** | **̶** | **◊** |
|  | *Polypodium polypodioides* (L.) Watt var. *polypodioides* | **○** | ***** | **̶** | **◊** | **̶** |
|  | *Polypodium* sp. 1 | **◊** | **̶** | **̶** | **◊** | **̶** |
|  | *Serpocaulon falcaria* (Kunze) A.R. Sm. | **̶** | **̶** | **̶** | **̶** | **○** |
|  | *Serpocaulon fraxinifolium* (Jacq.) A.R. Sm. | **̶** | **̶** | **̶** | ***** | ***** |
|  | *Terpsichore atroviridis* (Copel.) A.R. Sm. | **̶** | **̶** | **̶** | ***** | **̶** |
|  | *Terpsichore* sp. 1 | **̶** | **̶** | ***** | **̶** | **̶** |
| **Pteridaceae** | *Ananthacorus angustifolius* (Sw.) Underw. & Maxon | **̶** | ***** | **̶** | **̶** | **̶** |
|  | *Polytaenium feei* (W. Schaffn. ex Fée) Maxon | **̶** | **̶** | ***** | **̶** | **̶** |
|  | *Polytaenium* sp.1 | **̶** | **̶** | **◊** | **̶** | **̶** |
| **Selaginellaceae** | *Selaginella flagellata* Spring |  |  |  | **◊** | **◊** |
|  | *Selaginella* sp. 1 | **̶** | **◊** | **◊** | **◊** | **̶** |
| **Tectariaceae** | *Megalastrum biseriale* (Baker) A.R. Sm. & R.C. Moran | **̶** | **̶** | **◊** | **̶** | **̶** |
|  | *Tectaria heracleifolia* (Willd.) Underw. | **̶** | **̶** | **̶** | **◊** | **̶** |
|  | *Tectaria* sp*.* 1 | **◊** | **̶** | **̶** | **̶** | **̶** |
| **Thelypteridaceae** | *Thelypteris gigantea* (Mett.) R.M. Tryon | **̶** | **̶** | **̶** | **◊** | **̶** |
| **Vittariaceae** | *Vittaria graminifolia* Kaulf. | **̶** | **̶** | **̶** | ***** | **̶** |

**Table S3**

| **Parameters** | **TotalRain** | **MeanTemp** | **MeanLW** | **MeanRH** |
| --- | --- | --- | --- | --- |
| **TotalRain** | 1 |  |  |  |
| **MeanTemp** | 0.73 | 1 |  |  |
| **MeanLW** | -0.68 | -0.98 | 1 |  |
| **MeanRH** | -0.77 | -1 | 0.98 | 1 |

**Figure S1**


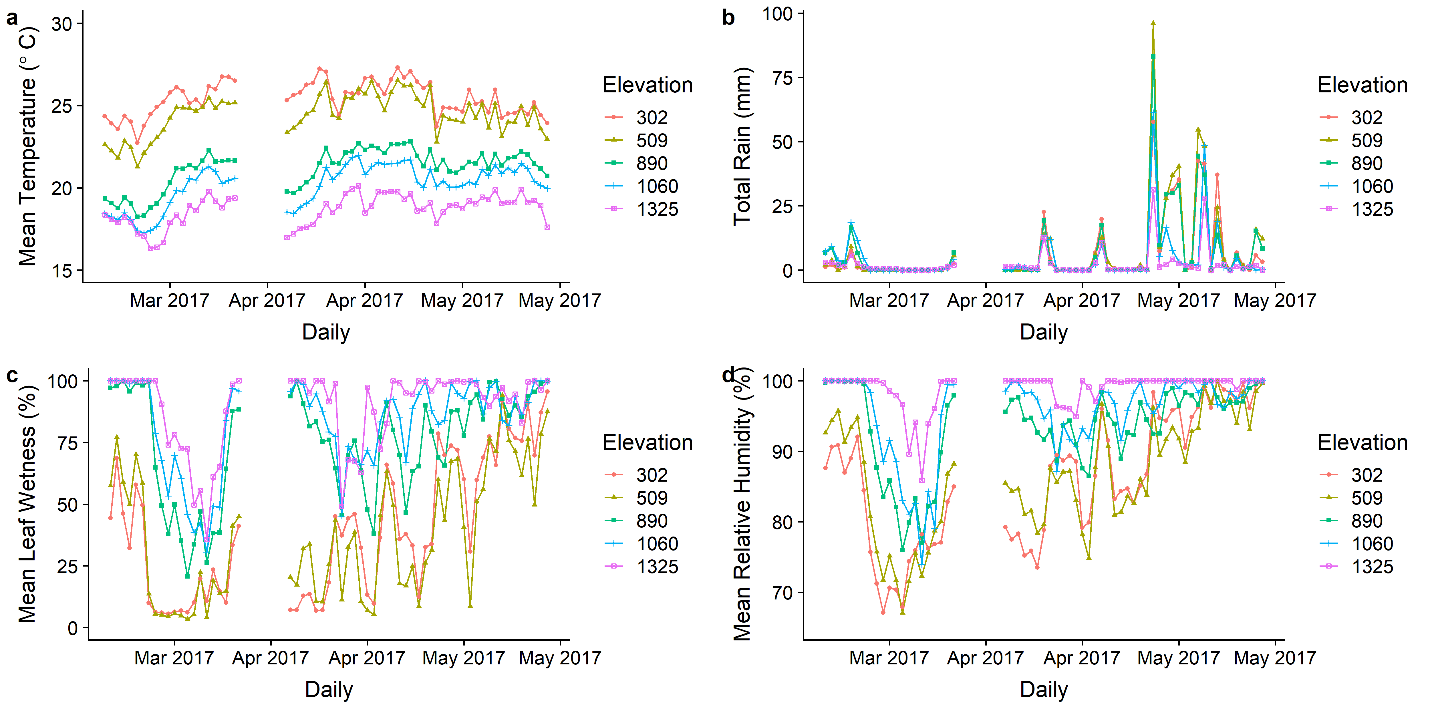


**Figure S2**


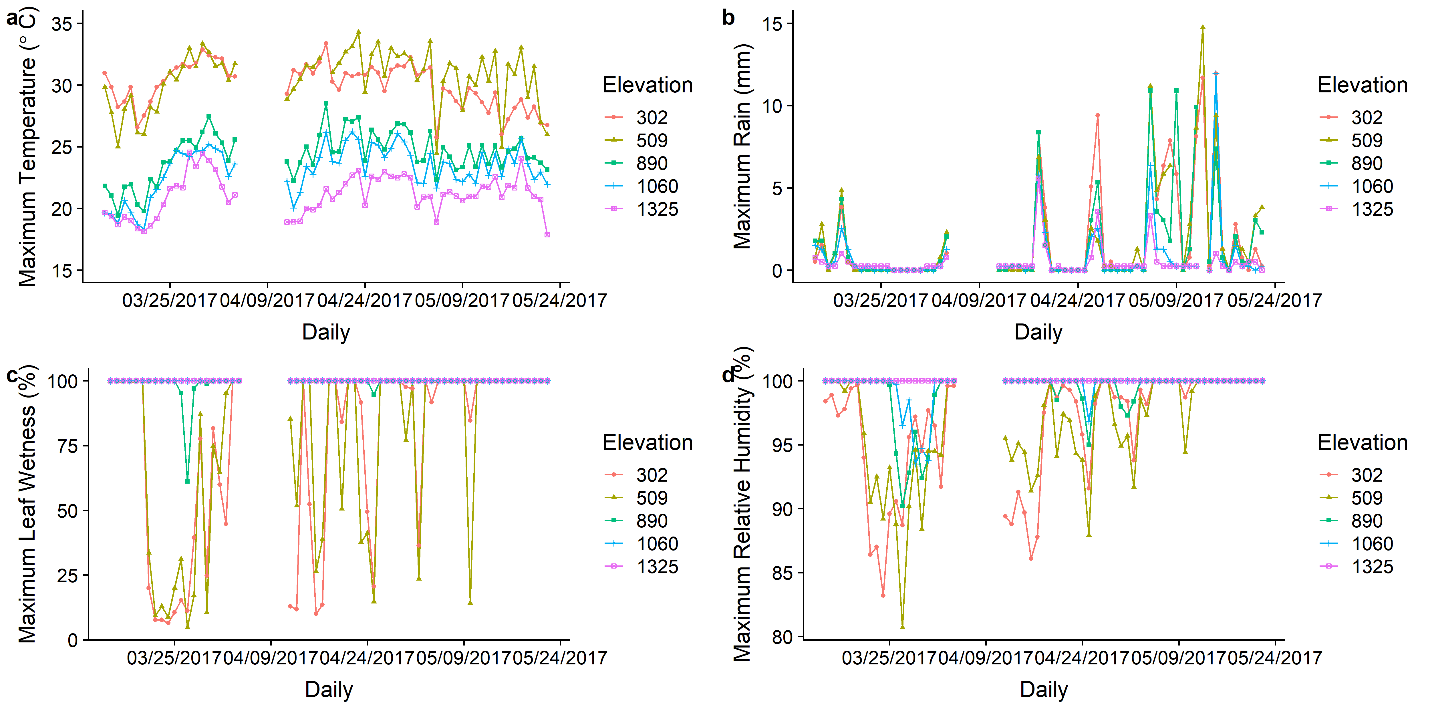


**Figure S3**


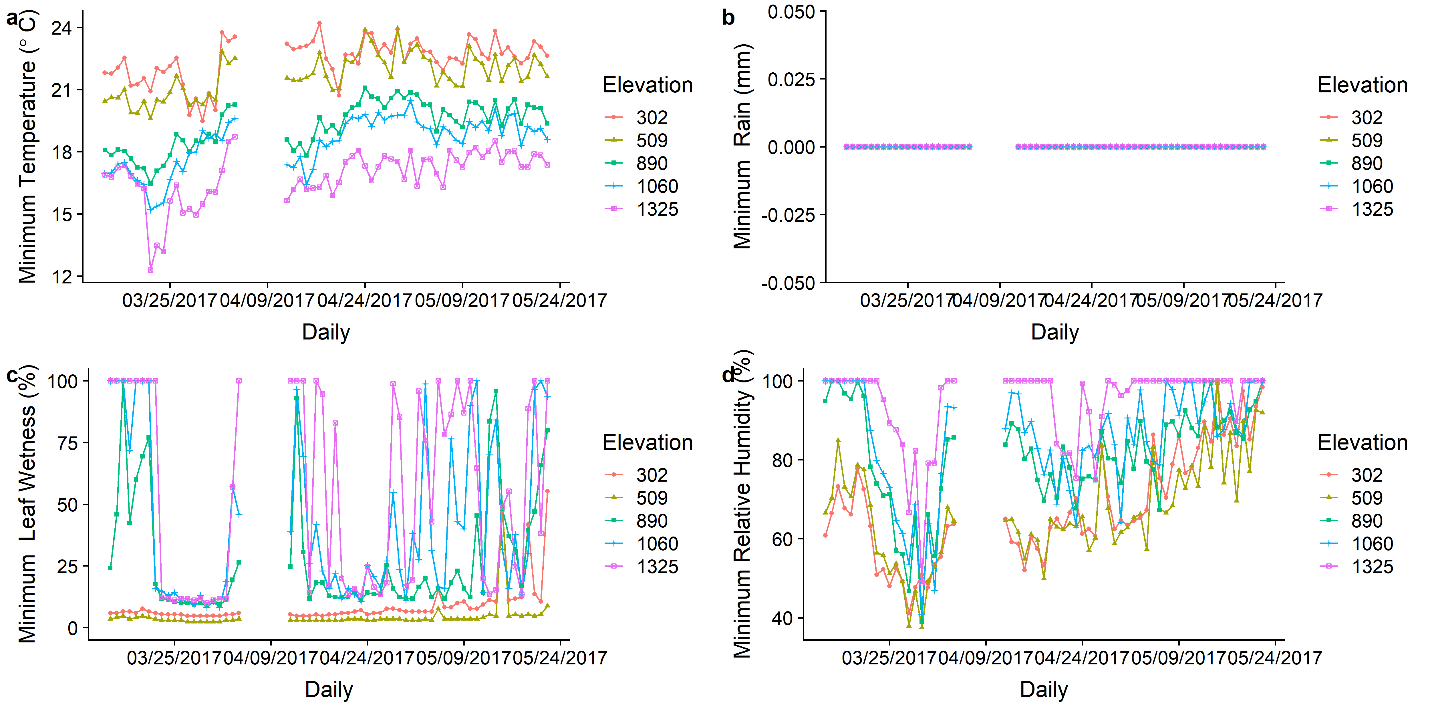


**Figure S4**


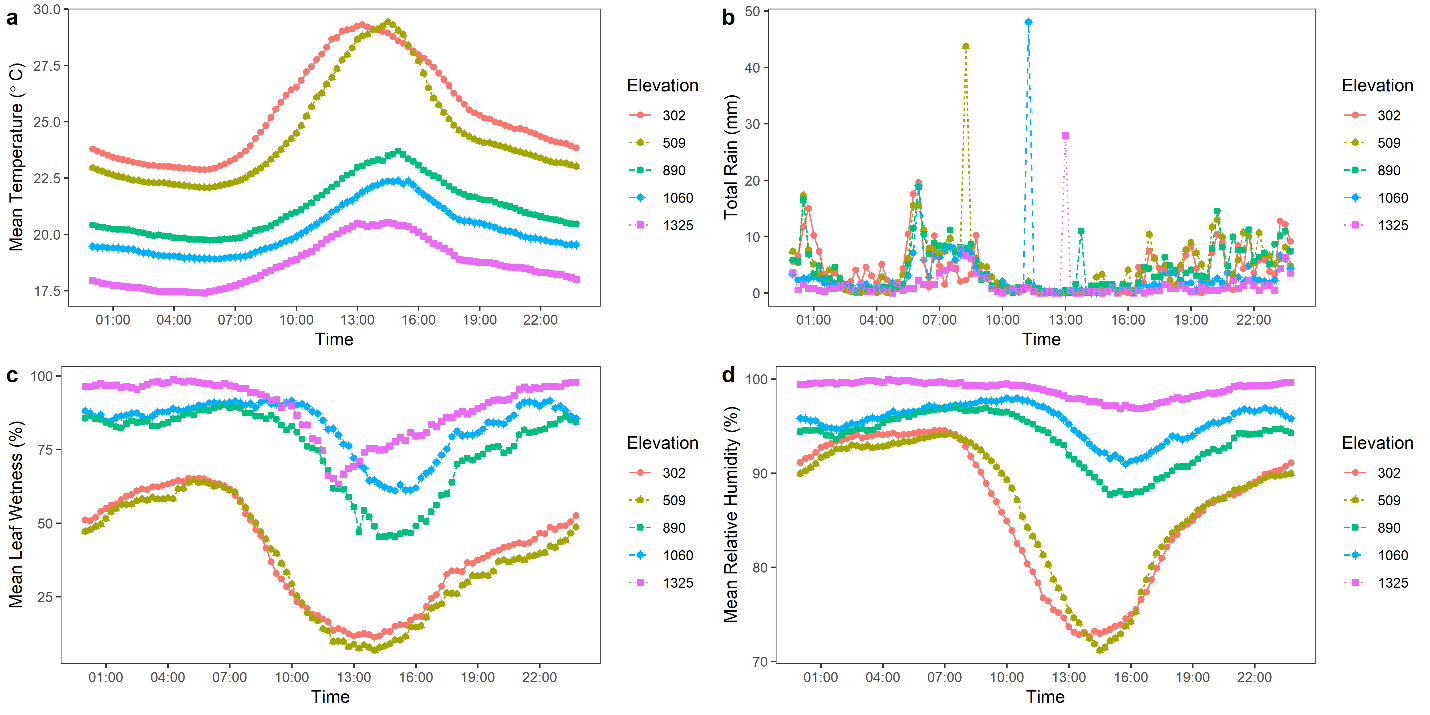

Supplement: Supplementary file 1 — Appendix S1 [file ECE3-12-e9501-s002.docx]
