# Supplementary material for: High species richness and turnover of vascular epiphytes is associated with water availability along the elevation gradient of Volcán Maderas, Nicaragua
Source: Ecol Evol. 2022 Nov 22;12(11):e9501. doi: 10.1002/ece3.9501 (PMC9682193; doi:10.1002/ece3.9501)
Supplement: Supplementary file 2 — Appendix S2 [file ECE3-12-e9501-s001.docx]

# Supplementary Material

**Table S1.** Total species richness of vascular epiphytes per family from the entire sampled elevational gradient of Volcán Maderas, Nicaragua. (DF=dry forest, HF=humid forest, WF=wet forest, CF=cloud forest, EF=elfin forest).

**Table S2.** Species composition and occurrence data per forest type (DF=dry forest, HF=humid forest, WF=wet forest, CF=cloud forest, EF=elfin forest). Points include data from sampled trees and fallen branches. A diamond shaped symbol (**◊**) represents presence of a vascular epiphyte or hemiepiphyte recorded by standarized approach (Ss). A star (*****) represents the presence of a vascular epiphyte or hemiephyte recorded by the opportunistic sampling (Os). An open circle (**○**) represents vascular epiphytes recorded by both the opportunistic and standardized sampling (Os + Ss).

**Table S3.** Correlation coefficients among all climatic variables measured with weather stations on Volcán Maderas, Nicaragua. MeanTemp = mean temperature (°C), MeanLW = mean leaf wetness (%), MeanRH = mean relative humidity (%).

**Figure S1.** Daily readings for (a) mean temperature (°C), (b) total rainfall (mm), (c) mean leaf wetness (%), and (d) mean relative humidity (%) during March 15, 2017- May 22, 2017. The different colored lines represent readings from the following elevations and forest types of Volcán Maderas, Nicaragua: 302 m=dry forest, 509 m=humid forest, 890 m=wet forest, 1060 m=cloud forest, 1325 m=elfin forest.

**Figure S2.** From the 96 climate (15-min interval) readings per day, the interval with the maximum value in a given day is shown for (a) temperature (°C), (b) rainfall (mm), (c) leaf wetness (%), and (d) relative humidity (%) during March 15, 2017- May 22, 2017. The different colored lines represent readings from the following elevations and forest type of Volcán Maderas, Nicaragua: 302 m=dry forest, 509 m=humid forest, 890 m=wet forest, 1060 m=cloud forest, 1325 m=elfin forest.

**Figure S3.** From the 96 climate (15-min interval) readings per day, the interval with the minimum value in a given day is shown for (a) temperature (°C), (b) rainfall (mm), (c) leaf wetness (%), and (d) relative humidity (%) during March 15, 2017- May 22, 2017. The different colored lines represent readings from the following elevations and forest type of Volcán Maderas, Nicaragua: 302 m=dry forest, 509 m=humid forest, 890 m=wet forest, 1060 m=cloud forest, 1325 m=elfin forest.

**Figure S4.** Readings for (a) mean temperature (°C), (b) total rainfall (mm), (c) mean leaf wetness (%), and (d) mean relative humidity (%), for a 24-hr. period. For all readings, mean values over the sampling period were taken for each of the 96, 15-minute daily time-points to show daily patterns. The different colored lines represent readings coming from the following elevations and forest types of Volcán Maderas, Nicaragua: 302 m=dry forest, 509 m=humid forest, 890 m=wet forest, 1060 m=cloud forest, 1325 m=elfin forest.
